# Rhodium(I) Complexes with a η^1^-Fluorenyl-*P*-phosphanylphosphorane Ligand

**DOI:** 10.1021/acs.inorgchem.4c01934

**Published:** 2024-07-18

**Authors:** Javier Eusamio, Nil Saumell, Anton Vidal-Ferran, Arnald Grabulosa

**Affiliations:** † Departament de Química Inorgànica i Orgànica, Secció de Química Inorgànica, Universitat de Barcelona, Martí i Franquès 1−11, E-08028 Barcelona, Spain; ‡ Institut de Nanociència i Nanotecnologia, Universitat de Barcelona, E-08028 Barcelona, Spain; § Institució Catalana de Rercerca i Estudis Avançats, Passeig Lluís Companys 23, E-08010 Barcelona, Spain

## Abstract

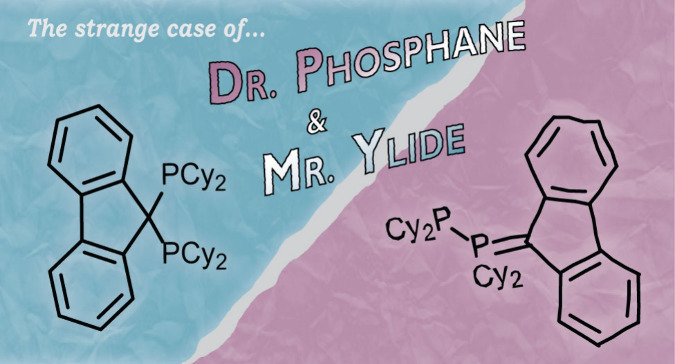

The first example of a *P*-phosphanylphosphorane, Flu=PCy_2_–PCy_2_ (**L2**; Flu = 9-fluorenyl), has been easily prepared by P-phosphination of lithiated 9-dicyclohexylphosphinofluorene (FluPCy_2_, **L0**) with chlorodicyclohexylphosphane. **L2** constitutes a new type of P(III)–P(V) organophosphorus compound, a σ^3^λ^3^–σ^4^λ^5^ species that is stable under an inert atmosphere in the solid state. The reaction of **L2** with [Rh(diene)_2_]BR_4_ causes metalation of the benzylic carbon (C9) of fluorene, giving κ^2^-C,P complexes in which fluorene is coordinated in the η^1^ form. A complex with the weakly coordinating BArF anion has been isolated and fully characterized, including its crystal structure obtained by X-ray diffraction.

## Abstract 2

Treatment of 9-dicyclohexylphosphinofluorene (FluPCy_2_, **L0**; Flu = 9-fluorenyl) with *n*-BuLi/PClCy_2_ has surprisingly yielded the P(III)−P(V) phosphane−phosphorane Flu=PCy_2_−PCy_2_ (**L2**), formed by P-phosphination, instead of the expected diphosphane, **L1**. Compound **L2** reacts with [Rh(diene)_2_]BR_4_, coordinates through the P(III) atom, and causes the metalation of C9 of fluorene, giving κ^2^-C,P complexes in which fluorene is coordinated in the η^1^ form.

Complexes with the fluorenyl ligand are significant because of their excellent performance in olefin polymerization with early transition metals.^1^ This is due to the versatility of the bonding of fluorenyl, encompassing η^5^, η^3^ and η^1^ coordination modes, easily interchanged during catalysis, a process known as ring slippage. Despite this, η^1^-coordination is relatively uncommon, even for late transition metals. A way to enforce it is by using of fluorenes with a coordinating side arm that modulates the steric and electronic properties of the ligand, producing cyclometalated complexes. For rhodium, only a handful of η^1^-fluorenyl complexes of this type have been structurally characterized (Figure 1, top).

**Figure 1:**
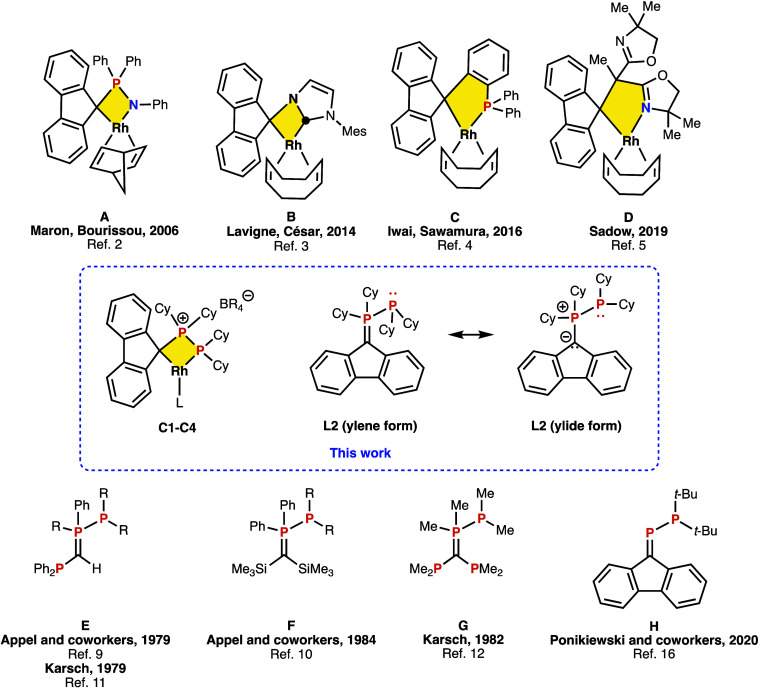
Rhodium complexes with a η^1^-fluorenyl (top, **A**–**D**), ligand **L2**, and derived complexes **C1**–**C4** presented in this work (middle) and examples of reported species related to **L2** (bottom, **E**–**H**).

Maron and Bourissou^2^ reported complex **A**, using a fluorenyl-phosphazene ligand coordinated in a κ^2^-C,N mode. Much later, Lavigne and César^3^ described **B**, with a N-heterocyclic carbene ligand coordinated in κ^2^-C,C fashion while Iwai and Sawamura^4^ studied triarylmethane–monophosphanes and reported **C**, bearing a fluorenyl-phosphane coordinated in a κ^2^-C,P fashion. Finally, Sadow^5^ described Rh complexes with bis(oxazolinyl)fluorenylphosphane, coordinated in a κ^2^-C,N fashion, such as **D**. In this contribution we describe rhodium(I) complexes **C1**–**C4**, with an unprecedented cyclometalated phosphanylphosphorane ligand **L2**, a P(III)-P(V) species (Figure 1, middle).

Compounds such as **L2** are unknown but other P(III)-P(V) species have been described.^6−8^ Some species related to **L2** are given in Figure 1 (bottom). Appel^9,10^ and simultaneously Karsch^11^ reported several *P*-phosphanylphosphoranes (Figure 1, compounds **E** and **F**). Slightly later, Karsh^12−14^ described tetraphosphorus compound **G**. Much more recently, Ponikiewski^15−18^ has reported phosphanylphosphaalkenes, like the fluorenyl-substituted compound,^19^**H**. In this contribution, we detail the unexpected^20^ synthesis of **L2**, its characterization and its coordination to rhodium(I).

Inagaki^20^ took advantage of the relative acidity of the methylene protons of fluorene to prepare 9,9-bis(di-R-phosphino)fluorenes (R = Cy, Ph). He described the synthesis of monophosphane **L0** (Scheme 1), without characterization details. We obtained it as a white solid in 90% yield, featuring a singlet at δ_P_ = +13.4 ppm in the ^31^P{^1^H} NMR spectrum. **L0** is sensitive to oxidation so it was boronated for storage (δ_P_ = +35.3 ppm), although in low yield.

**Scheme 1:**
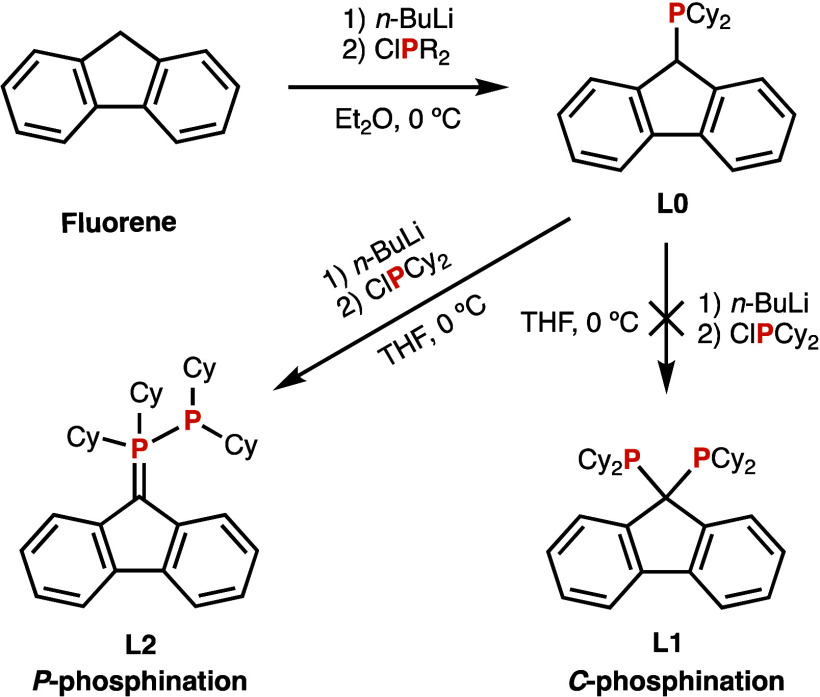
Attempted Synthesis of **L1** and Obtention of **L2**

Following our interest in methylene-bridged diphosphanes,^21,22^ we reasoned that fluorene should be an excellent platform for single-atom-bridged, electron-rich diphosphanes,^23,24^ and we attempted the synthesis^20^ of **L1** from **L0** (Scheme 1).

Unexpectedly, a different result was found when treating **L0** with *n*-BuLi/PClCy_2_, following the reported procedure.^20^ The ^31^P{^1^H} NMR spectrum contained two doublets at δ_P_ = +23.50 and −2.36 ppm with a coupling constant of 308.9 Hz. It was thought that the bulkiness and rigidity of the molecule could render the two dicyclohexylphosphino moieties of **L1** nonequivalent and coupled “through-space”^25^ but heating a sample up to 60 °C in benzene-*d*_6_ did not give any hints of coalescence either in ^1^H or ^31^P{^1^H} NMR spectra.

The very large *J*_P–P_ suggested a compound with a direct P–P bond, ruling out diphosphane **L1**, although the exact mass was the expected one [*m*/*z* 559.3672, matching with M(**L1**) – H], indicating that the product was an isomer of **L1**. In addition, in the ^13^C{^1^H} NMR spectrum a quaternary non aromatic carbon appears as a *doublet of doublets* (δ_C_ = 55.4 ppm; *J*_CP_ = 79.6, 3.5 Hz), which must correspond to the C9 carbon of fluorene. All these data are consistent with the formation of compound **L2** instead of **L1** (Scheme 1). **L2** can be described as a *P*-phosphanylphosphorane, with a double P–C bond. In the coordination number (σ)–valency (λ) nomenclature, **L2** is a σ^3^λ^3^–σ^4^λ^5^ species. Compound **L2** was obtained in a 60% yield as a pearl white solid, which is stable in the solid state under an inert atmosphere. In contrast, when the solid was exposed to air for a few days, NMR spectroscopy showed that it decomposed giving **L0** (δ_P_ = +13.4 ppm) and the known secondary phosphane oxide HP(O)Cy_2_ (δ_P_ = +49.5 ppm)^26^ together with other species. The decomposition is accelerated by light. The same reaction has been observed for related compounds and has been attributed to hydrolysis.^9^

The formation of **L2** occurs by an unexpected P-phosphination instead of the reported C-phosphination of the carbanion derived from **L0**. The formation of an α-carbanion increases the electron density of the phosphorus atom. In the case of **L0** this effect and the cyclohexyl groups makes the phosphorus so nucleophilic that C-phosphination is completely suppressed and **L1** is not formed. This was observed by Appel^9,10^ (**E** and **F** in Figure 1) and very recently Rufanov^27^ reported the P-alkylation of 9-fluorenyldiphenylphosphane with alkyl halides, although the reaction with chlorodiphenylphosphane was in the carbon, as reported by Inagaki.^20^

The attention was then turned to the complexation of **L2** to see whether the P–C double bond would remain upon coordination.^28^ Treatment of [Rh(nbd)_2_]BF_4_ with 1 equiv of **L2** gave a red solid after workup (**C1**; Scheme 2).

**Scheme 2:**
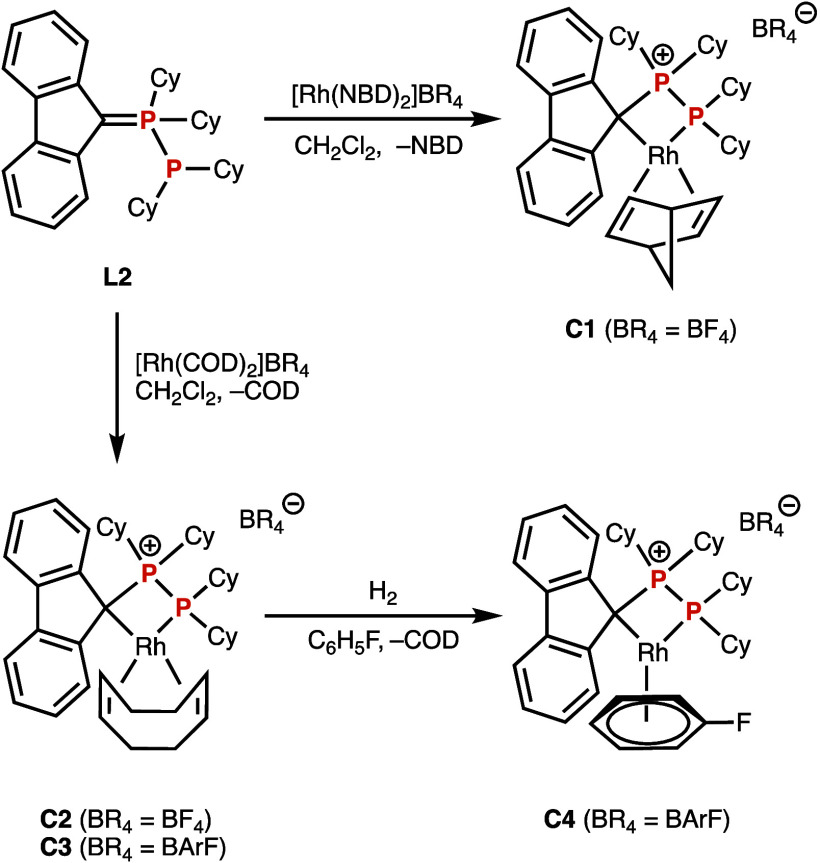
Complexation of Phosphanephosphorane **L2** to Rhodium(I) Moieties

The ^31^P{^1^H} NMR spectrum of **C1** (Figure S1, top) presented two doublets of doublets, at δ_P_ = +88.7 (*J* = 163.6 and 21.5 Hz) and −19.7 ppm (*J* = 163.6 and 144.7 Hz). The strong shielding with respect to **L2** is typical for the formation of four-membered phosphametallacycles and is due to the ring contribution (Δ_R_).^29^ We encountered it in complexes of methylene-bridge diphosphanes.^21,22^

Since the reactivity of [Rh(diene)_2_]X (X = weakly coordinating anion) can depend on the diene,^30,31^**L2** was reacted with [Rh(cod)_2_]BF_4_. Compound **C2** was obtained, but it was impurified with unidentified species. Finally, the weakly coordinating anion can be noninnocent,^32^ and given our experience with the tetrakis[3,5-bis(trifluoromethyl)phenyl]borate (BArF) anion,^33−37^ we reacted **L2** with [Rh(cod)_2_]BArF. In this case, the reaction was cleaner, giving complex **C3** in a pure form after recrystallization. This complex presents two doublets of doublets in the ^31^P{^1^H} NMR spectrum (Figure S1, bottom) at δ_P_ = +88.5 (*J* = 178.7 and 18.5 Hz) and −31.8 ppm (*J* = 178.6 and 136.9 Hz).

The ^31^P{^1^H} NMR spectra of **C1**–**C3** clearly show that the P–P structure remains after coordination, with a ^2^*J*_PP_ of 178.7 Hz for **C3**. The more shielded signal must correspond to the P(III) of the dicyclohexylphosphino moiety coordinated to rhodium, with a standard ^1^*J*_PRh_ of 136.9 Hz for **C3**. However, the peak at lower fields has a much smaller *J*_PRh_ of 18.5 Hz, which can not correspond to a species with a direct P–Rh bond. Another interesting feature of **C3** are the chemical shifts of the cod unsaturated groups. In the ^1^H NMR spectrum, they appear as two broad singlets at δ_H_ = 4.64 and 2.47 ppm, integrating two protons each. The latter chemical shift is extremely low for alkene protons, even considering that cod is coordinated to rhodium. This suggests that one of the double bonds of cod is affected by the aromatic ring currents of the π electrons of the fluorenyl group, causing a strong shielding.^38^ In addition, the 2D ^1^H–^13^C{^1^H} NMR HSQC spectrum shows that while the sp^2^ carbon atoms of cod bound to the deshielded protons appear, in the ^13^C{^1^H} NMR spectrum, as the expected doublet (δ_C_ = 82.7 ppm; *J*_CRh_ = 9.0 Hz), the carbons of the shielded protons resonate as a triplet (δ_C_ = 91.4 ppm; *J* = 10.4 Hz). This can only be explained by a coupling with rhodium and phosphorus with a similar constant, suggesting that one of the cod double bonds is trans to a phosphorus atom, but not the other.

All these data shows that activation of the P–C double bond of **L2** has occurred so **C1**–**C3** are cyclometalated rhodium(I) compounds, having a Rh–C bond with the bis(benzylic) carbon C9 of fluorene (Scheme 2).

It is interesting to note that in complexes **C1**–**C3** ligand **L2** acts as bidentate ligand in a κ^2^-C,P fashion. In other words, **L2** can be formally viewed as a zwitterionic *P*-phosphanylphosphonium fluorenide ligand that uses the C–P double bond electron pair for coordination, as the right resonance (mesomeric) structure shows (Figure 1, middle). This octet rule compliant structure should be the predominant one.

To produce a complex with a more labile ligand, **C3** was dissolved in fluorobenzene and pressurized with 4 bar of hydrogen, to substitute the cod ligand by fluorobenzene^39^ and give complex **C4** (Scheme 2). After 1 h, ^31^P{^1^H} NMR spectroscopy indicated only 10% conversion.^40^ Several attempts were carried out at longer reaction times, but **C3**/**C4** mixtures with many other peaks in the ^31^P{^1^H} NMR spectrum were invariably obtained and for long reaction times, rhodium black appeared.

Complexes **C1**–**C4** turned out to be very stable and a concentrated fluorobenzene solution of the **C3**/**C4** 90:10 mixture spontaneously yielded a crop of beautiful, dark red cubic crystals that were analyzed by X-ray diffraction.^41^

The asymmetric unit contains one molecule of the complex and one BArF anion. The rhodium atom is coordinated to a mixture cyclooctadiene (85%) and fluorobenzene (15%). Figure 2 displays the two different metal cations that can be extracted from the crystal structure, and Table S1 gives the more informative geometric parameters.

**Figure 2:**
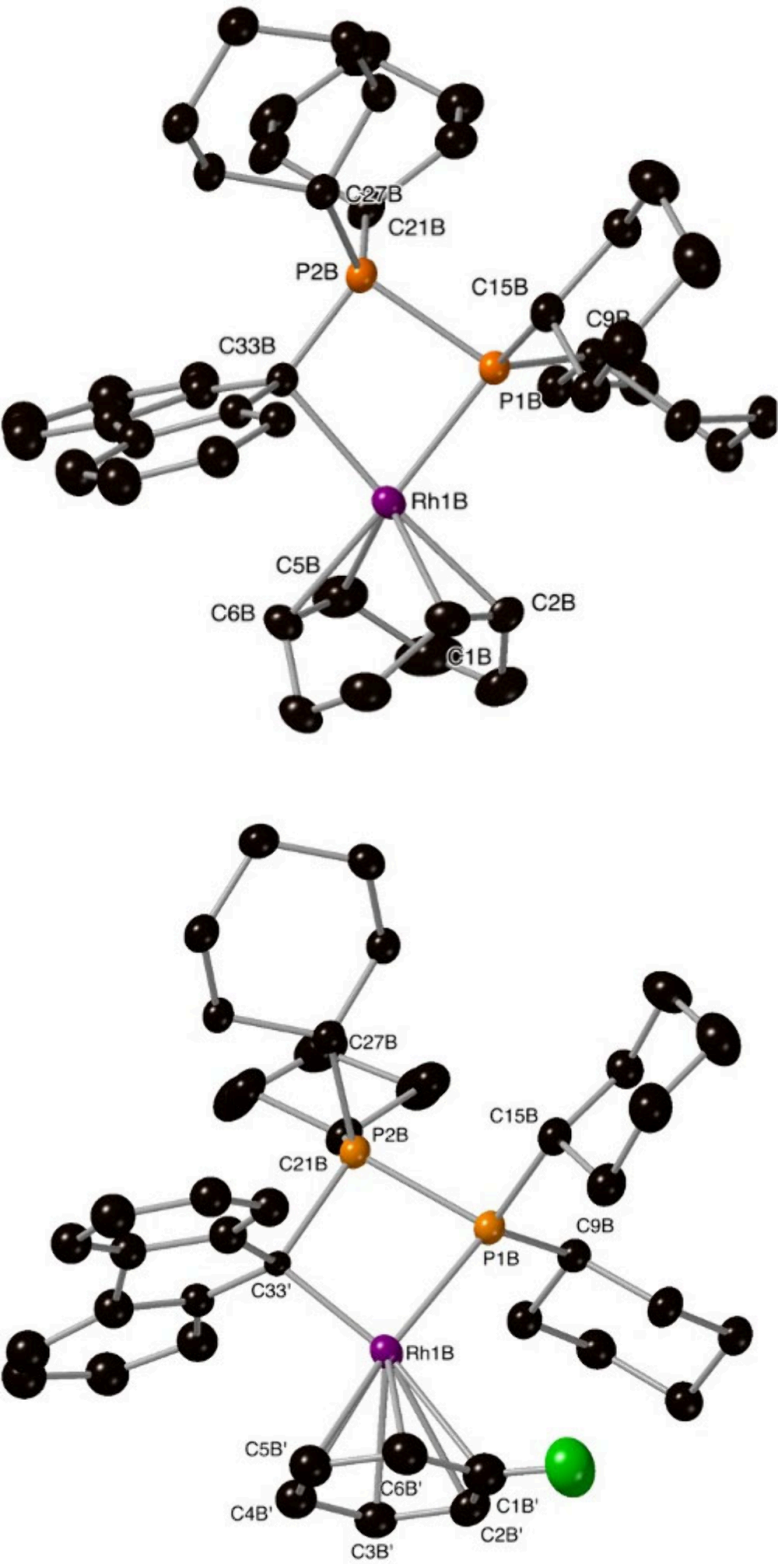
Ellipsoid plot of the cations of **C3** (top) and **C4** (bottom) with ellipsoids drawn at the 50% probability level. Hydrogen atoms and BArF anions have been omitted for clarity.

The Rh1B–P1B(Cy_2_)–P2B(Cy_2_) fragment is crystallographically identical in the two cations, but they differ in the coordinated fluorenyl ligand and obviously in the ancillary ligand (cod for **C3** or fluorobenzene for **C4**).

Both structures show that the rhodium(I) center is coordinated to the P1B phosphorus of the phosphane group of **L2** and to fluorene, coordinated in a σ,η^1^ fashion, by carbon C33B or C33′, which corresponds to C9 in the standard numbering of fluorene. In the cod cation, the two double bonds of cod are η^2^,η^2^-coordinated to Rh, as expected, completing the expected square-planar geometry of the metal. In the fluorobenzene cation, the arene is coordinated in a η^6^ fashion, giving, formally, a pentacoordinate geometry around the rhodium center.

**L2** acts as a bidentate, κ^2^-C,P-coordinated ligand, giving an essentially planar Rh1B–P1B–P2B–C33B/C33′ four-membered ring for both cations of Figure 2. There are, however, differences because in **C3** (Figure 2, top) the distance Rh1B–C33B [2.212(4) Å] is considerably longer than the distance Rh1B–C33′ [2.012(15) Å] of **C4** (Figure 2, bottom) and the opposite happens with the C33B/C33–P2B distances. As a consequence, ligand **L2** has a smaller bite angle in **C3** [79.84(11)°] than in **C4** [85.6(4)°]. This can be due to the bulkier (tridimensional) nature of cod compared to 2D fluorobenzene, causing a larger value of the Rh–fluorene distance in **C3** compared to **C4**. In addition, the fluorenyl substituent is much more planar in the complex with cod than in the complex with fluorobenzene. All of these differences are not unexpected because formally **C3** is a tetracoordinated, square-planar, 16e^–^ complex, while **C4** is a pentacoordinated, 18e^–^ complex.

In conclusion, P-phosphination of **L0** has produced the *P*-phosphanylphosphorane **L2**, whose coordination to [Rh(diene)_2_]BR_4_ has given cyclorhodated complexes, containing a η^1^-coordinated fluorenyl ligand. 9-Fluorenyl is an electron-releasing, sterically demanding substituent^42^ that has produced phosphanes for catalysis.^43−45^ The metalation of fluorene presented here should further increase electron donation and steric crowding to the metal. Additionally, it is likely that similar compounds with other substituents should be accessible. We are currently studying this in our laboratories.

## Supporting Information Available

Figure S1, Table S1, full experimental details (no uncommon hazards are noted), and NMR, IR and MS spectra of the new compounds (PDF)
