# Supplementary material for: Rhodium(I) Complexes with a η1-Fluorenyl-P-phosphanylphosphorane Ligand
Source: Inorg Chem. 2024 Jul 18;63(30):13820–4. doi: 10.1021/acs.inorgchem.4c01934 (PMC11289756; doi:10.1021/acs.inorgchem.4c01934)
Supplement: Supplementary file 1 — ic4c01934_si_001.pdf [file ic4c01934_si_001.pdf]

Supporting Information for:

Rhodium(I) Complexes with a η^1-Fluorenyl, *P*-Phosphanyl-Phosphorane Ligand

Javier Eusamio,[a,b] Nil Saumell,[a] Anton Vidal-Ferran,[a,b,c] and Arnald Grabulosa[a,b,*]

[a]Departament de Química Inorgànica i Orgànica, Secció de Química Inorgànica, Universitat de Barcelona, Martí i Franquès, 1-11, E-08028, Barcelona, Spain. [b]Institut de Nanociència i Nanotecnologia (IN2UB), Universitat de Barcelona, E-08028, Barcelona, Spain. [c]Institució Catalana de Rercerca i Estudis Avançats (ICREA), Passeig Lluís Companys 23, E-08010, Barcelona, Spain.

Table of contents

Experimental part **S1**

Figure S1 .... **S6**

Table S1 .... **S6**

Spectra of **L0** **S7**

Spectra of **L0·BH$_3$** **S13**

Spectra of **L2** **S20**

Spectra of **C1** **S29**

Spectra of **C3** **S35**

Experimental part

Generalities

All compounds were prepared under a purified nitrogen atmosphere using standard Schlenk and vacuum-line techniques or inside a glovebox. No uncommon hazards are noted. The solvents were purified by a solvent purification system or by standard procedures[1] and kept under nitrogen. ^{1}H, $^{13}C\{^{1}H\}$, $^{31}P\{^{1}H\}$, and bidimensional NMR spectra were recorded at room temperature with 400 or 500 MHz spectrometers. The fields are 400 or 500 MHz (^{1}H), 101 or 126 MHz ($^{13}C\{^{1}H\}$) and 162 or 202 MHz ($^{31}P\{^{1}H\}$). Chemical shifts are reported downfield from standards ($SiMe_4$ for ^{1}H and ^{13}C and H_3PO_4 for ^{31}P NMR) and the coupling constants are given in Hz. IR spectra were recorded with an ATR and the main absorption bands are expressed in cm^{-1}. High-resolution mass analyses (HRMS) were carried out in a time-of-flight instrument using electrospray ionisation.

Ligands

L0, 9-(Dicyclohexylphosphino)fluorene (FluPCy$_2$)

This compound was prepared by lithiation of fluorene and phosphination with $PClCy_2$ in 90 % yield, following the reported procedure of Inagaki and coworkers.[2] Since the characterisation was not reported, it is given here.

^{1}H NMR (400 MHz, C_6D_6): 7.72–7.69 (m, 4H, Ar), 7.29–7.21 (m, 4H, Ar), 4.33 (s, 1H, $H_{H9(Flu)}$), 1.66–1.63 (m, 12H, Cy), 1.14-0.97 (m, 10H, Cy). **^{13}C{^{1}H} NMR (101 MHz, C_6D_6)**: 146.5 (d, J_{CP} = 3.0, C, Ar), 141.3 (s, C, Ar), 126.9 (d, J_{CP} = 7.1, CH, Ar), 125.5 (d, J_{CP} = 6.1, CH, Ar), 43.1 (d, J_{CP} = 30.3, CH, $CH_{Flu(C9)}$), 31.3 (d, J_{CP} = 14.1, CH), 31.0 (d, J_{CP} = 14.1, CH_2), 27.5 (d, J_{CP} = 9.1, CH_2), 27.5 (d, J_{CP} = 10.1, CH_2), 26.7 (s, CH_2). **^{31}P{^{1}H} NMR (162 MHz, C_6D_6)**: +13.4 (s). **HRMS**: calcd. for $[M + H]^+$ 363.2236, found 363.2250. **IR**: 2918, 2845, 1442, 786, 733.

L0·BH₃, 9-(Dicyclohexylphosphino)fluorene-borane (FluPCy₂·BH₃)

Fluorene (500 mg, 2.86 mmol) was dissolved in 20 mL of diethyl ether and the solution was cooled to 0 °C. *n*-BuLi (2.06 mL of a 1.6 M solution, 3.3 mmol) was added dropwise, the mixture was stirred for 30 min and $PClCy_2$ (0.74 mL, 784 mg, 2.2 mmol) was added. The mixture was stirred for 14 h at room temperature, giving a yellow suspension that was brought to dryness. The crude was washed with methanol (3 x 4 mL), dissolved in 20 mL of THF and to the yellow solution a 1 M solution of BH_3·THF (6 mL, 6 mmol) was added. After stirring for 1 h, the solvents were evacuated, and the crude was extracted with dichloromethane (3 x 20 mL) and water. The combined organic phase was dried with anhydrous sodium sulfate, filtered, and brought to dryness under vacuum, yielding thick yellow solid. The solid was recrystallized in dichloromethane/ethanol, giving a whitish powder. Yield: 34 mg (9 %).

^{1}H NMR (400 MHz, $CDCl_3$): 7.82–7.77 (m, 2H, Ar), 7.45–7.42 (m, 2H, Ar), 7.35–7.31 (m, 2H, Ar), 4.59 (d, J = 12.0, 1H, $H_{H9(Flu)}$), 1.84-1.62 (m, 13H), 1.28-1.02 (m, 12H). **^{13}C{^{1}H} NMR (101 MHz, $CDCl_3$)**: 141.4 (d, C, J_{CP} = 3.0, Ar), 141.3 (d, C, J_{CP} = 4.0, Ar), 128.0 (s, CH, Ar), 126.9 (s, CH, Ar), 126.5 (d, CH, J_{CP} = 2.0, Ar), 120.1 (s, CH, Ar), 42.3 (d, J_{CP} = 21.3, CH, $CH_{Flu(C9)}$), 31.2 (d, J_{CP} = 28.3, CH), 27.2-27.0 (m, CH_2), 25.9 (s, CH_2). **^{31}P{^{1}H} NMR (162 MHz, $CDCl_3$)**: +35.2 (br). **HRMS**: calcd. for $[M – BH_3 + H]^+$ 363.2236, found 363.2236. **IR**: 2927, 2844, 2389 (ν(B–P)), 2341 (ν(B–P)), 1443, 1064, 885, 797, 732, 599.

L2, (Flu=PCy₂–PCy₂)

L0 (362 mg, 1.0 mmol) was dissolved in 5 mL of THF, and the solution was cooled to 0 °C. *n*-BuLi (0.7 mL of a 1.6 M solution, 1.1 mmol) was carefully added. The mixture was stirred for 30 min and a solution of $PClCy_2$ (0.26 mL, 275 mg, 1.2 mmol) in 5 mL of THF was added dropwise and the solution is left stirring for 14 h. The resulting suspension was brought to dryness under vacuum, washed with diethyl ether (3 x 4 mL) at 0 °C and dried under vacuum to yield the title product. Yield: 334 mg (60 %).

^{1}H NMR (400 MHz, C_6D_6): 8.45 (d, *J* = 7.6 Hz, 2H, Ar), 7.82 (d, *J* = 8.4 Hz, 2H, Ar), 7.59-7.55 (m, 2H, Ar), 7.34 (t, *J* = 7.0 Hz, 2H, Ar), 3.49-3.40 (m, 2H), 2.43 (br, 4H, CH), 2.20-2.11 (m, 2H), 1.87-0.60 (m, 36H). **^{13}C{^{1}H} NMR (101 MHz, C_6D_6)**: 140.6 (d, J_{CP} = 13.3, C, Ar), 132.3 (d, J_{CP} = 12.7, C, Ar), 123.7 (s, CH, Ar), 120.5 (s, CH, Ar), 117.a (s, CH), 116.6 (s, CH, Ar), 55.4 (dd, $^1J_{CP}$ = 79.6, $^3J_{CP}$ = 3.5, $C_{Flu(C9)}$), 39.0 (d, J_{CP} = 9.4,

CH), 38.7 (d, J_{CP} = 9.3, CH), 34.3 (d, J = 6.8, CH), 34.3 (d, J_{CP} = 6.8, CH), 32.7 (d, J_{CP} = 3.0, CH_2), 32.4 (d, J_{CP} = 3.0, CH_2), 31.3 (d, J_{CP} = 6.5, CH_2), 31.2 (d, J_{CP} = 6.5, CH_2), 30.4 (d, J_{CP} = 3.4, CH_2), 30.4 (d, J_{CP} = 3.4, CH_2), 29.9 (d, J_{CP} = 4.3, CH_2), 28.4 (d, J_{CP} = 12.5, CH_2), 28.3 (d, J_{CP} = 12.4, CH_2), 28.0 (d, J_{CP} = 13.7, CH_2), 27.3 (d, J_{CP} = 7.0, CH_2), 26.6 (s, CH_2), 26.3 (s, CH_2). **$^{31}P\{^1H\}$ NMR (162 MHz, C_6D_6)**: +25.8 (d, J_{PP} = 308.9 Hz), –0.6 (d, J = 308.9 Hz). **HRMS**: calcd. for $[M – H]^+$ 559.3617 $C_{37}H_{53}P_2^+$; found 559.3638. **IR**: 2917, 1438, 1252, 1177, 1119, 746.

Rhodium complexes

C1, [Rh(nbd)(L2)]BF_4

In the glovebox, $[Rh(nbd)_2]BF_4$ (34.4 mg, 0.09 mmol) was dissolved in 1.6 mL of dichloromethane in a 10 mL amber scintillation vial. In a separate vial, **L2** was weighted (50.3 mg, 0.09 mmol) and 1.5 mL of DCM were added, forming a suspension. The suspension was transferred to the vial with the metal precursor and left stirring for 1 h. After that, the resulting suspension was filtered through a pad of Celite, brought to dryness, and recrystallized with dichloromethane/hexane. The desired product was obtained as red crystals (53 mg, 70 %).

^1H NMR (400 MHz, CD_2Cl_2): 8.05 (d, J = 8.0, 2H, Ar), 7.75 (d, J = 8.0, 2H, Ar), 7.50 (td, J = 7.2, 1.6, 2H, Ar), 7.40 (t, J = 7.6, 2H, Ar), 5.21 (br, 1H), 3.85 (br, 1H), 3.70-3.62 (m, 2H), 3.35 (br, 2H), 2.66 (br, 2H), 2.38 (br, 2H), 2.38-0.97 (m, 42H). **$^{13}C\{^1H\}$ NMR (101 MHz, CD_2Cl_2)**: 136.1 (d, J_{CP} = 6.9, C, Ar), 135.3 (C, Ar), 126.2 (CH, Ar), 124.5 (CH, Ar), 121.4 (CH, Ar), 117.8 (CH, Ar), 64.4 (CH_2, nbd), 51.4 (CH, nbd), 40.0 (d, J_{CP} = 5.1, CH), 37.0 (d, J_{CP} = 3.5, CH), 32.6-23.0 (m, CH_2). **$^{31}P\{^1H\}$ NMR (162 MHz, CD_2Cl_2)**: +88.7 (*dd*, $^1J_{PP}$ = 163.6, $^2J_{PRh}$ = 21.5), –19.7 (*dd*, $^1J_{PP}$ = 163.6, $^1J_{PRh}$ = 144.7). **HRMS**: calcd. for $[M – BF_4]^+$ 753.3220, found 753.3198. **IR**: 2922, 2850, 1444, 1058, 1002, 759, 739, 517,496.

C3, [Rh(cod)(L2)]BArF

The same procedure for complex **C1** was followed, using $[Rh(cod)_2]$BArF (106 mg, 0.09 mmol) as a metal precursor. The crude was recrystallized in fluorobenzene/hexane, affording a very crystalline red solid (124 mg, 84 %).

^1H NMR (400 MHz, CD_2Cl_2): 8.03–8.01 (m, 1H, Ar), 7.77–7.72 (m, 13H, Ar), 7.56 (br, 4H, Ar), 7.50–7.42 (m, 4H, Ar), 4.64 (br, 2H), 3.54-3.47 (m, 2H), 2.62 (br, 2H), 2.47 (br,

2H), 2.25-1.27 (m, 46H). **$^{13}C\{^1H\}$ NMR (101 MHz, CD_2Cl_2)**: 162.2 (q, J_{CB} = 50.1, C_{BArF}), 137.4 (d, J_{CP} = 6.6, C), 135.2 (CH_{BArF}), 137.4 (d, J_{CP} = 6.6, C, Ar), 130.4 (d, J_{CP} = 7.9, C, Ar), 129.2 (qm, J_{CF} = 32.0, C_{BArF}), 126.5-126.2 (CH, Ar), 122.1 (CH, Ar), 117.9 (m, CH_{BArF}), 117.3 (CH, Ar), 115.5 (d, J_{CP} = 21.0, C), 91.4 (t, J = 10.4, CH), 82.7 (d, J_{CRh} = 9.0, CH), 40.3 (d, J_{CP} = 4.3, CH), 37.5 (d, J_{CP} = 4.3, CH), 32.1-26.0 (m, CH_2). **$^{31}P\{^1H\}$ NMR (162 MHz, CD_2Cl_2)**: +88.5 (*dd*, $^1J_{PP}$ = 178.7, $^2J_{PRh}$ = 18.5), –31.9 (*dd*, $^1J_{PP}$ = 178.7, $^1J_{PRh}$ = 136.9). **HRMS**: calcd. for $[M - BArF]^+$ 769.3533, found 769.3532. **IR**: 2940, 2860, 1610, 1449, 1352, 1273, 1112, 883, 714, 682.

Single crystal X-ray structure determination of C3/C4

Crystal preparation. Crystals of **C3/C4** were grown from a concentrated solution of in fluorobenzene. The measured crystals were prepared under inert conditions immersed in perfluoropolyether as protecting oil for manipulation.

Data collection. Crystal structure determination of **C3/C4** was carried out using an Apex DUO Kappa 4-axis goniometer equipped with an APPEX 2 4K CCD area detector, a Microfocus Source E025 IuS using MoK_α radiation (0.71073 Å), Quazar MX multilayer Optics as monochromator and an Oxford Cryosystems low temperature device Cryostream 700 plus (T = –173 °C). Full-sphere data collection was used with ω and φ scans. *Programs used:* Data collection APEX-2,[3] data reduction Bruker Saint[4] V/.60A and absorption correction SADABS.[5]

Structure Solution and Refinement. Crystal structure solution was achieved using the computer program SHELXT.[6] Visualization was performed with the program SHELXle.[7] Missing atoms were subsequently located from difference Fourier synthesis and added to the atom list. Least-squares refinement on F^2 using all measured intensities was carried out using the program SHELXL 2015.[8] All non-hydrogen atoms were refined including anisotropic displacement parameters.

Comment to the structure C3/C4. The asymmetric unit contains one molecule of the metal complex and one BArF anion. In the main molecule the metal atom is coordinated to a mixture of two different ligands (cyclooctadiene and fluorobenzene). The ratio of ligands is cyclooctadiene 85 % and fluorobenzene 15 %. Also, the aromatic system bound to rhodium and phosphorus is disordered in two different orientations. In the BArF anion some of the CF_3-groups show rotational disorder.

References

(1) Armarego, W. L. F. *Purification of Laboratory Chemicals*; Butterworth Heinemann, 8th Ed., 2017.

(2) Matsusaka, Y.; Shitaya, S.; Nomura, K.; Inagaki, A. Synthesis of Mono-, Di-, and Trinuclear Rhodium Diphosphine Complexes Containing Light-Harvesting Fluorene Backbones. *Inorg. Chem.* **2017**, *56,* 1027-1030.

(3) Data collection with APEX II v2014.9-0. Bruker (2014). Bruker AXS Inc., Madison, Wisconsin, USA

(4) Data reduction with Bruker SAINT+ version V8.35A. Bruker (2013). Bruker AXS Inc., Madison, Wisconsin, USA.

(5) Blessing, R. H. An Empirical Correction for Absorption Anisotropy. *Acta Cryst.* **1995**, *A51,* 33-38.

(6) Sheldrick, G. M. Crystal structure refinement with *SHELX*. *Acta Cryst., Sect. C: Struct. Chem.* **2015**, *C71,* 3-8.

(7) Hubschle, C. B.; Sheldrick, G. M.; Dittrich, B. ShelXle: a Qt graphical user interface for *SHELXL*. *J. Appl. Cryst.* **2011**, *44,* 1281-1284.

(8) Sheldrick, G. SHELXT - Integrated space-group and crystal-structure determination. *Acta Cryst., Sect. A* **2015**, *71,* 3-8.

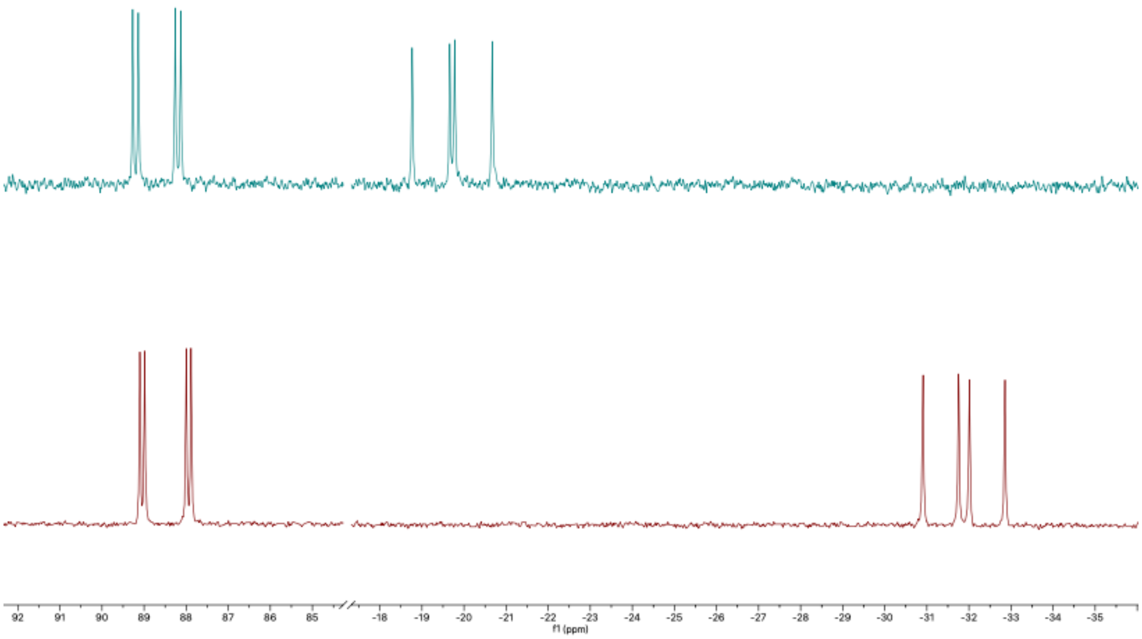

Figure S1. $^{31}P\{^1H\}$ NMR spectra (CD_2Cl_2, 162 MHz) **C1** (top) and **C3** (bottom).

Table S1. Selected distances (Å) and angles (deg) of the cations of **C3** and **C4**.

Parameter[a]	***C3***	***C4***
P1B-P2B	2.283(11)	
Rh1B-P1B	2.3105(10)	
Rh1B-C33B/C33’	2.212(4)	2.012(15)
P2B-C33B/C33’	1.765(4)	2.014(15)
P1B-Rh1B-C33B/C33’	79.84(11)	85.6(4)
Rh1B-C33B/C33’-P2B	101.55(17)	100.4(6)
C33B/C33’-P2B-P1B	92.55(14)	87.8(4)
P2B-P1B-Rh1B	85.92(3)	
[a]C33 for **C3** and C33’ for **C4**.		

$^{31}P\{^1H\}$ NMR (162 MHz, C_6D_6)

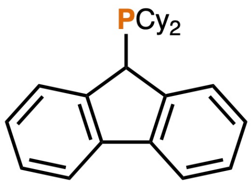

L0

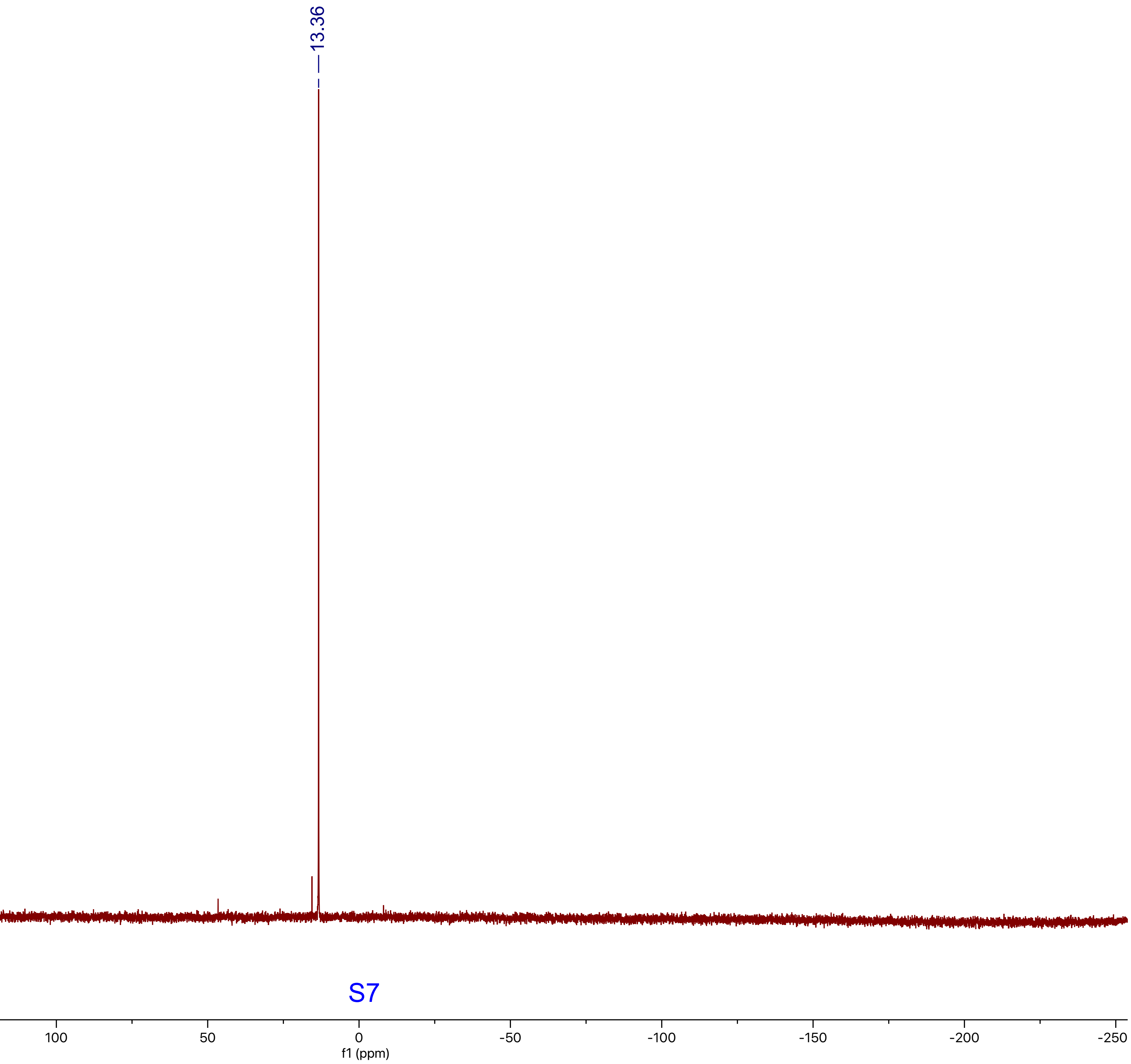

^{1}H NMR (400 MHz, C_6D_6)

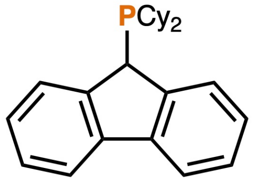

L0

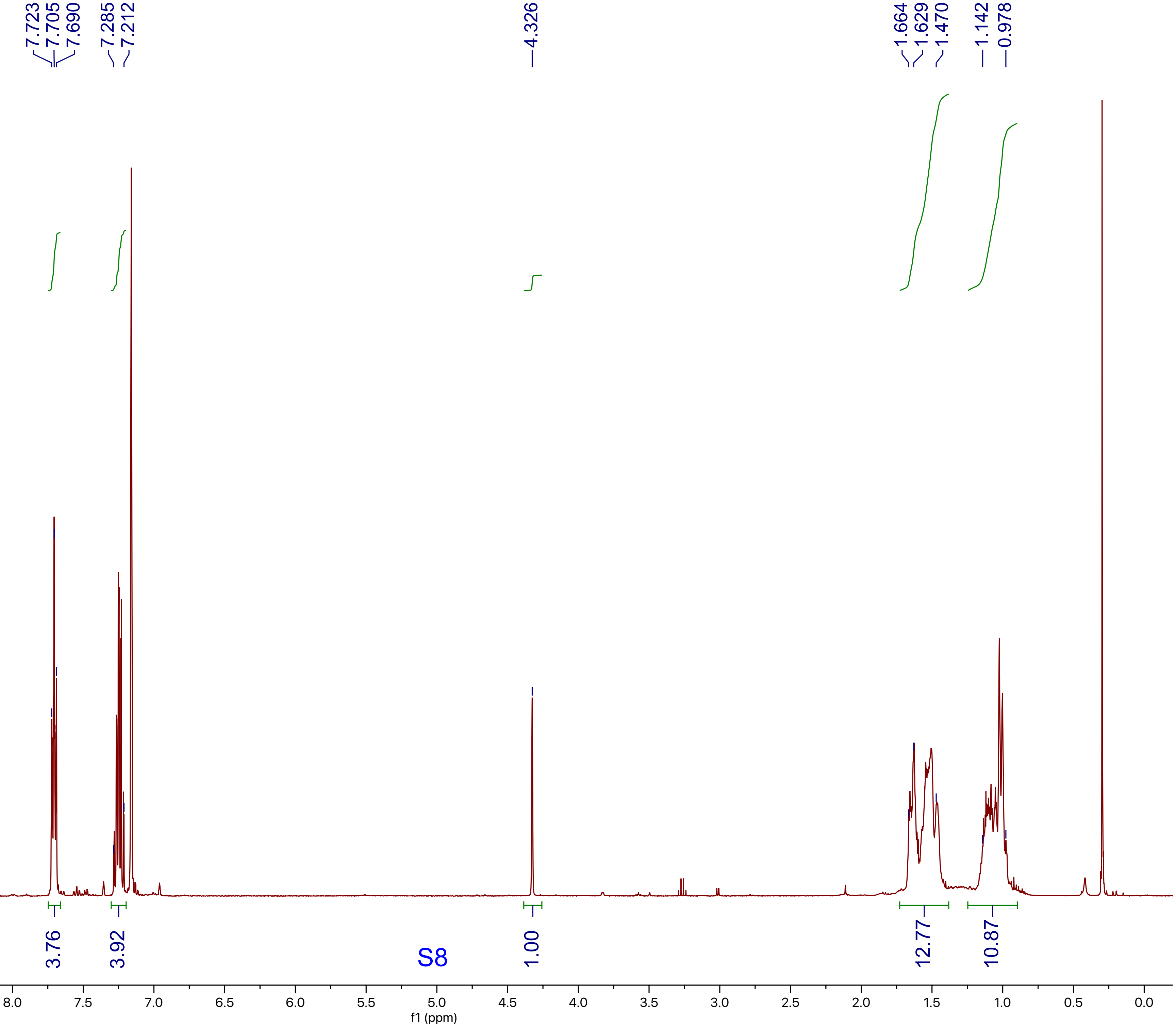

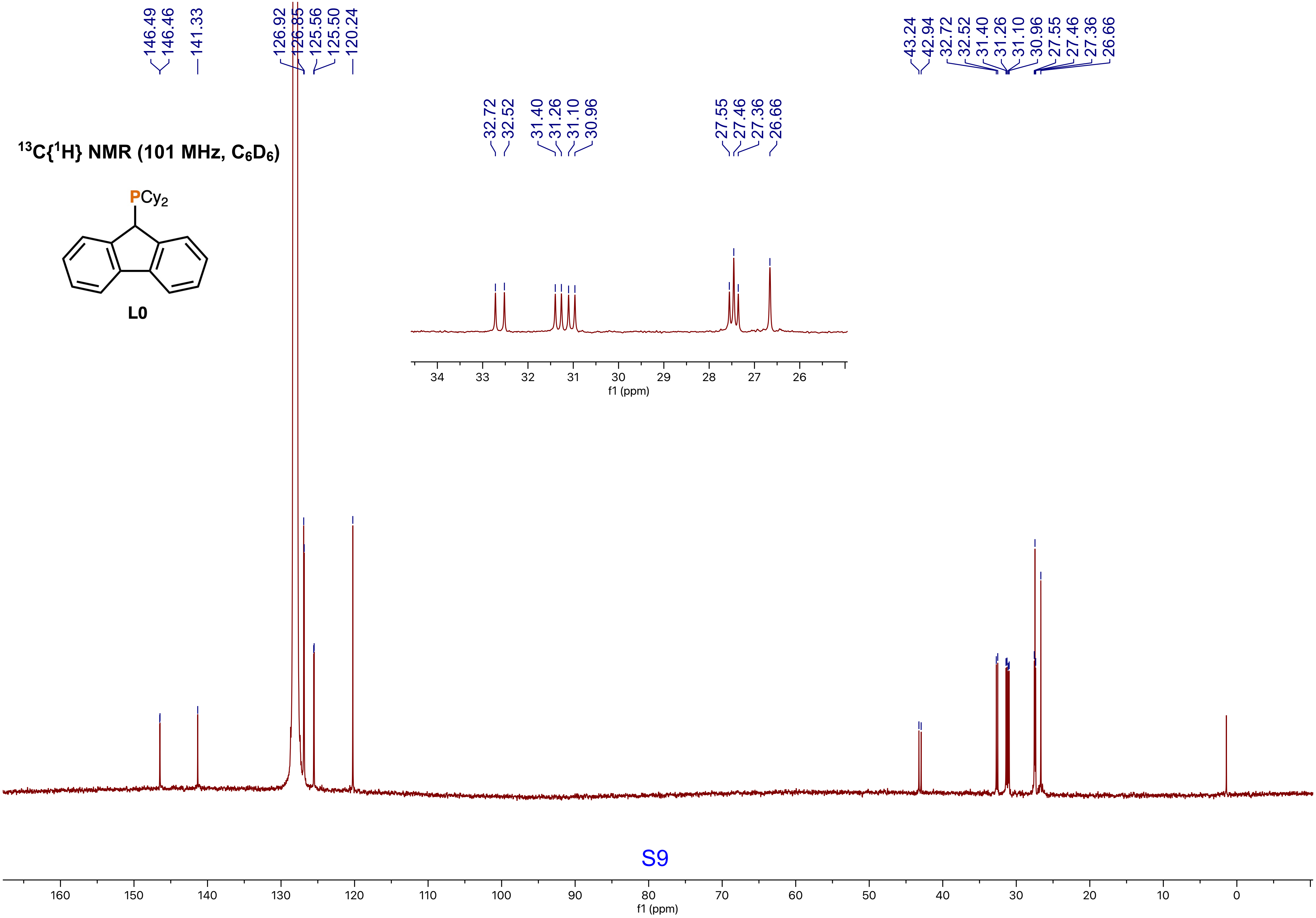

146.49
146.46
141.33
126.92
126.85
125.56
125.50
120.24
43.24
42.94
32.72
32.52
31.40
31.26
31.10
30.96
27.55
27.46
27.36
26.66
13C{1H} NMR (101 MHz, C6D6)
PCy2
L0
34 33 32 31 30 29 28 27 26
f1 (ppm)
160 150 140 130 120 110 100 90 80 70 60 50 40 30 20 10 0
f1 (ppm)

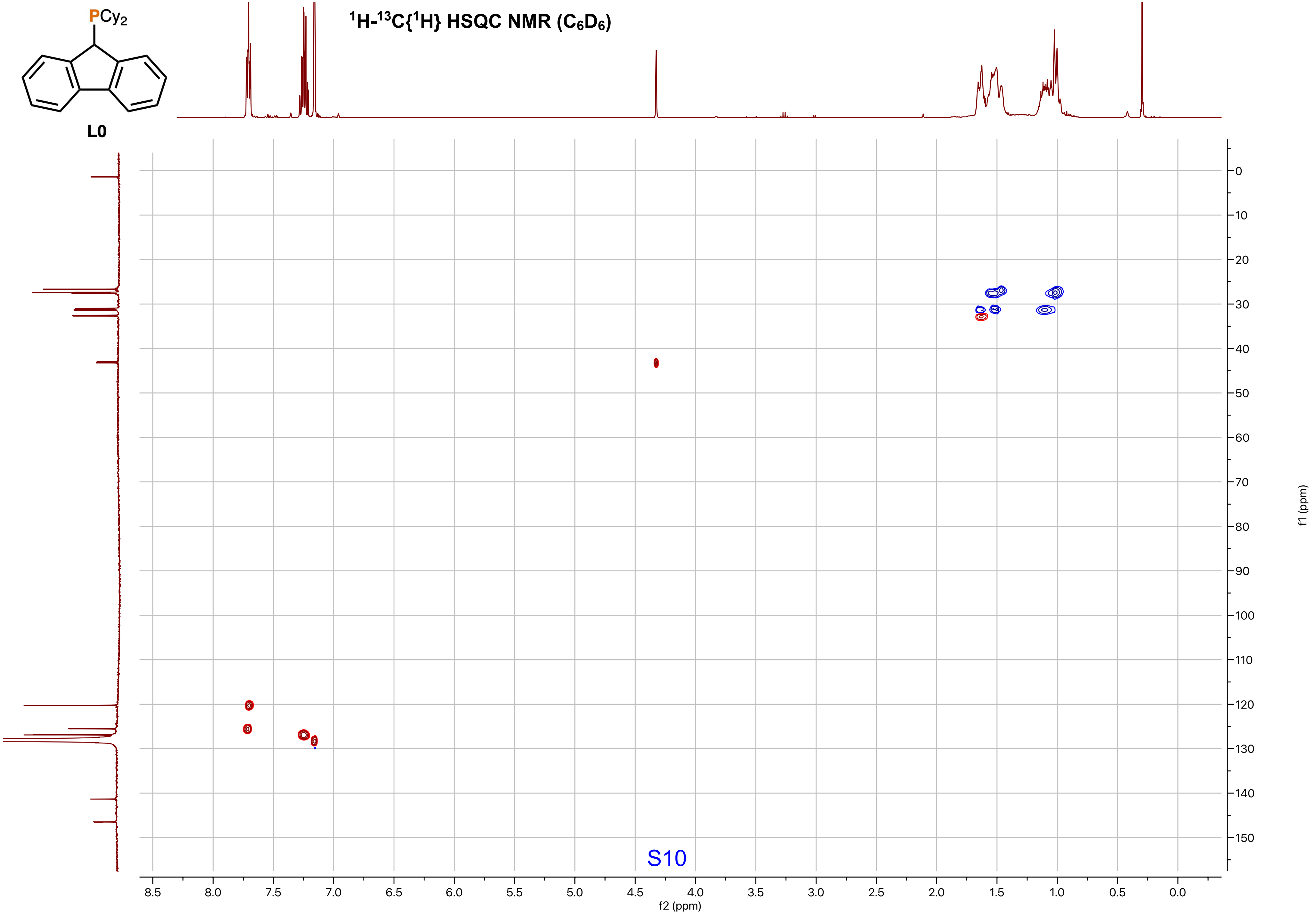

^{1}H-$^{13}C\{^{1}H\}$ HSQC NMR (C_6D_6)

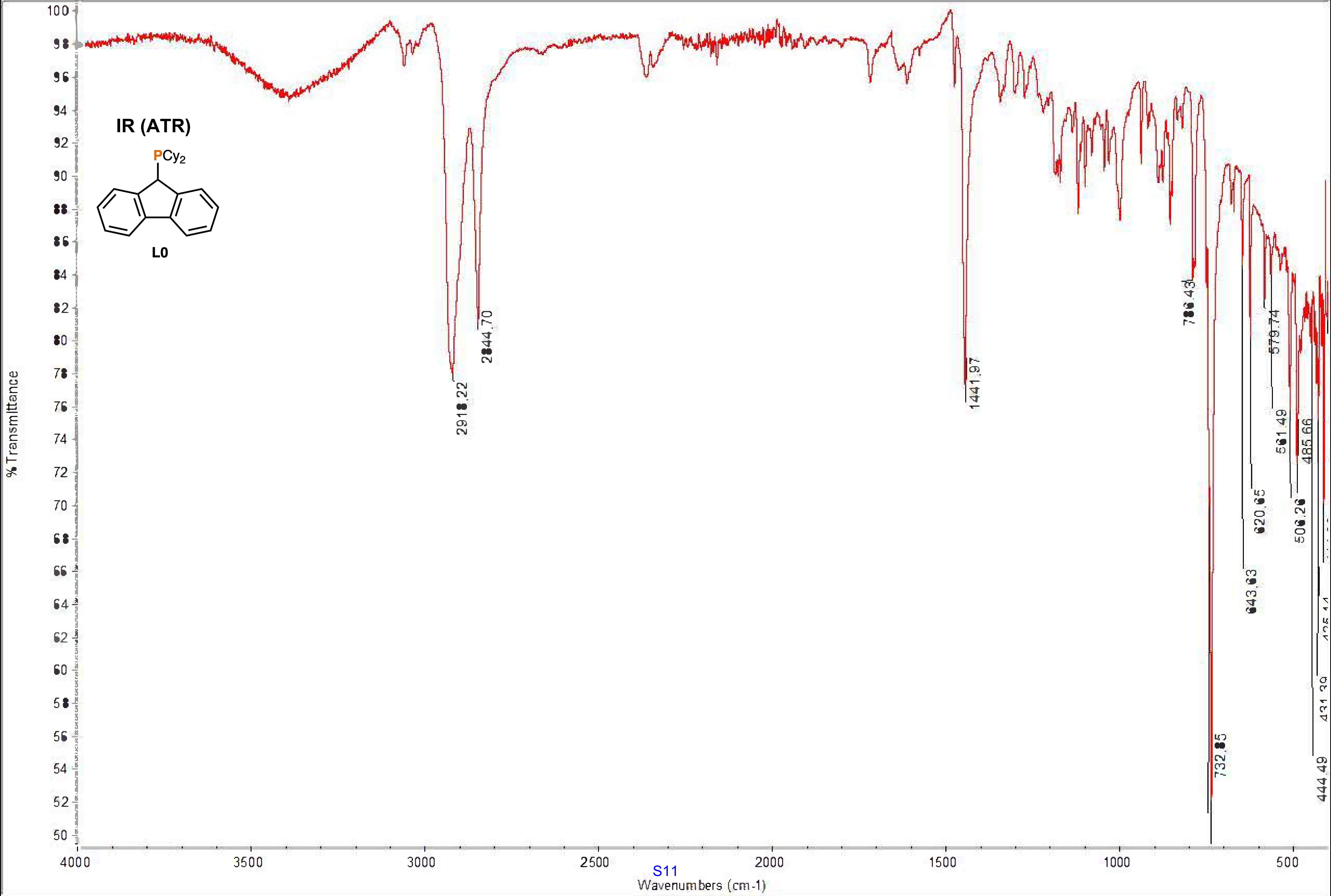
IR (ATR)
PCy2
L0
2918.22
2844.70
1441.97
786.43
732.85
643.63
620.65
579.74
561.49
506.26
485.66
444.49
%Transmittance
Wavenumbers (cm-1)

HRMS

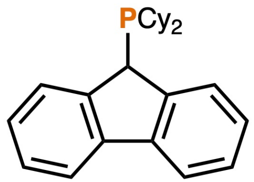
PCy2

L0

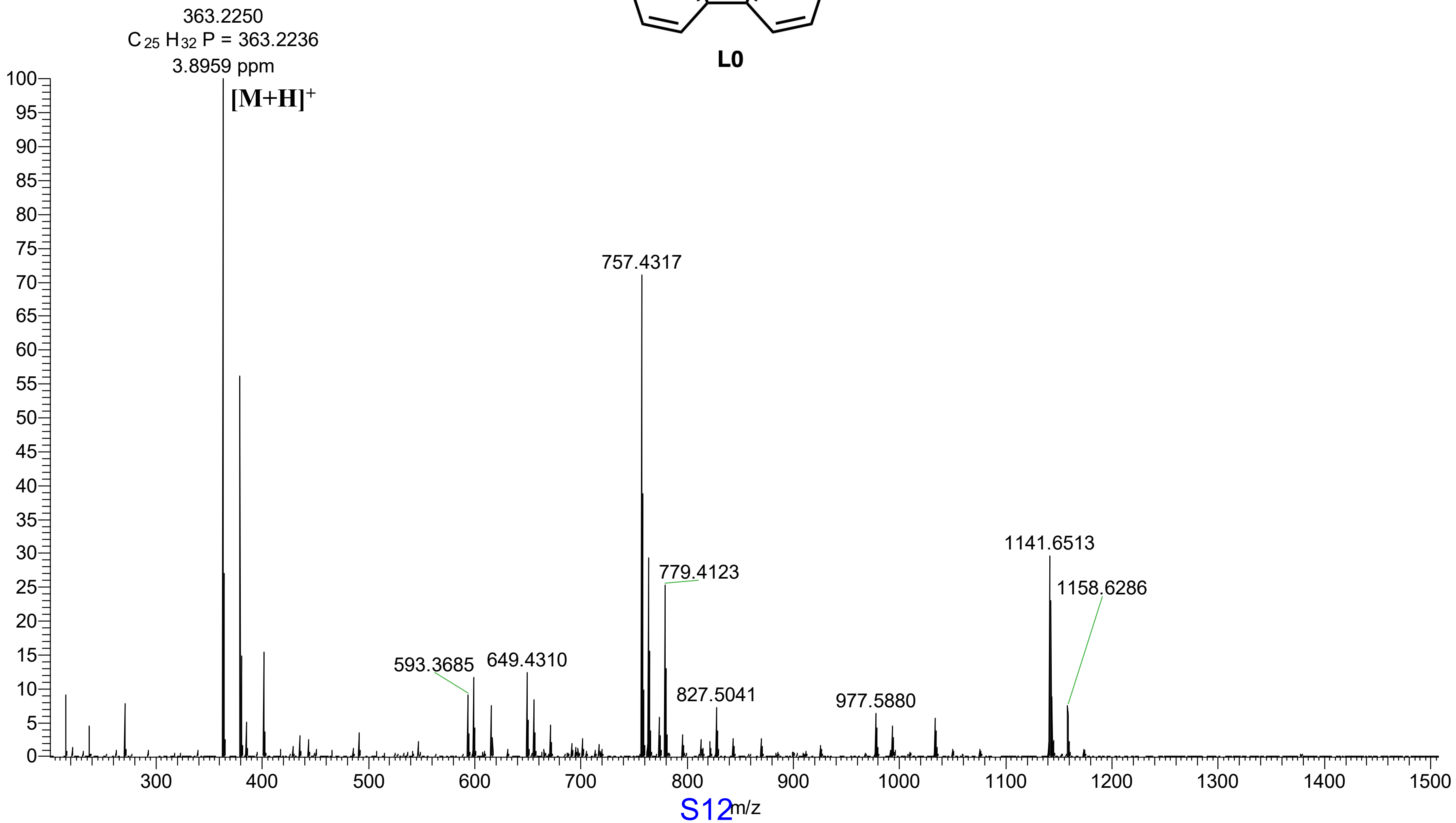
363.2250
C 25 H32 P = 363.2236
3.8959 ppm
[M+H]+
757.4317
779.4123
593.3685
649.4310
827.5041
977.5880
1141.6513
1158.6286
m/z

$^{31}P\{^1H\}$ NMR (162 MHz, $CDCl_3$)

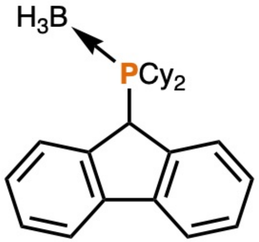

L0·BH$_3$

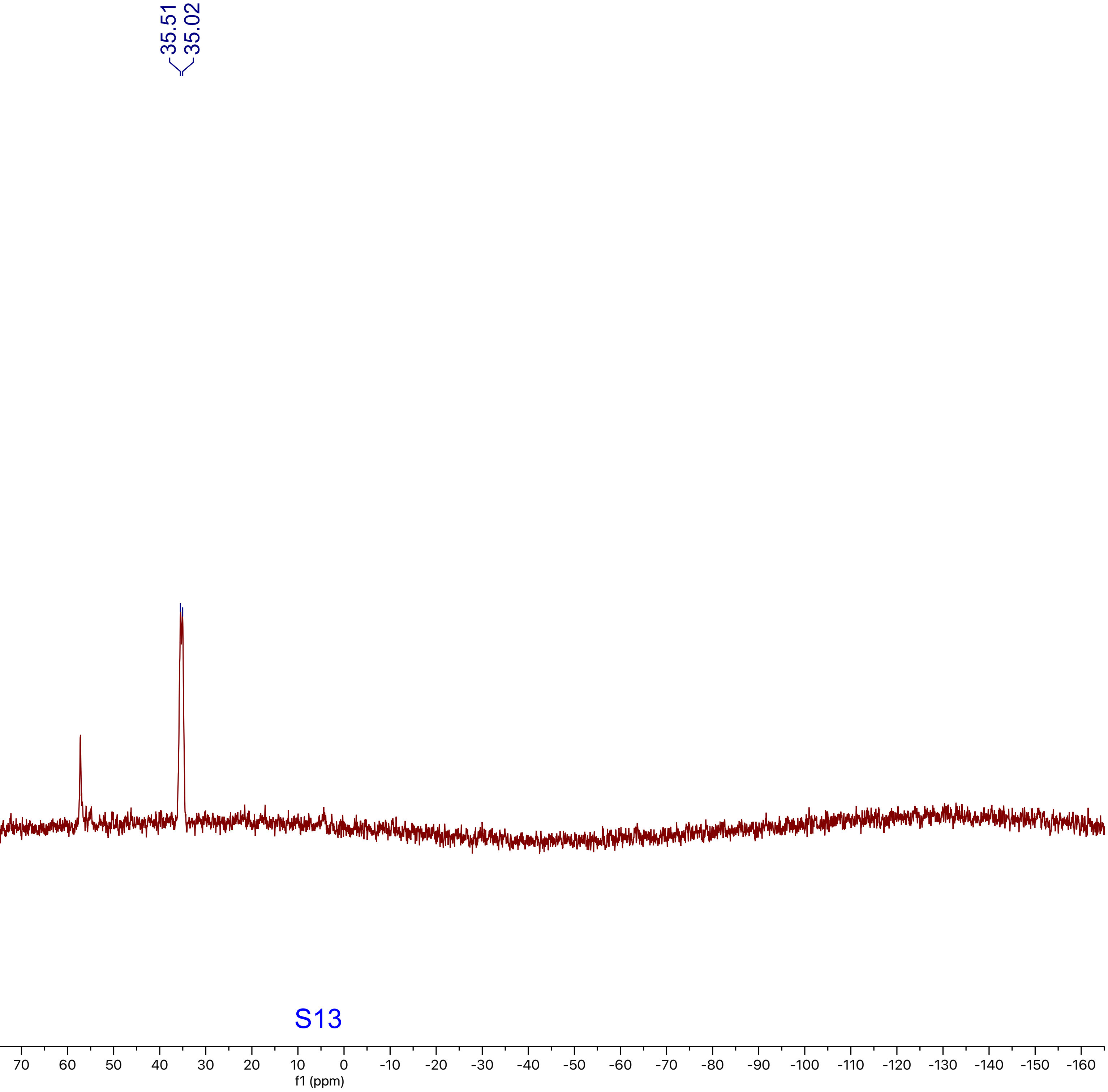

^{1}H NMR (400 MHz, $CDCl_3$)

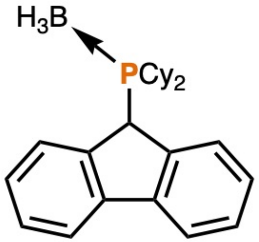

L0·BH_3

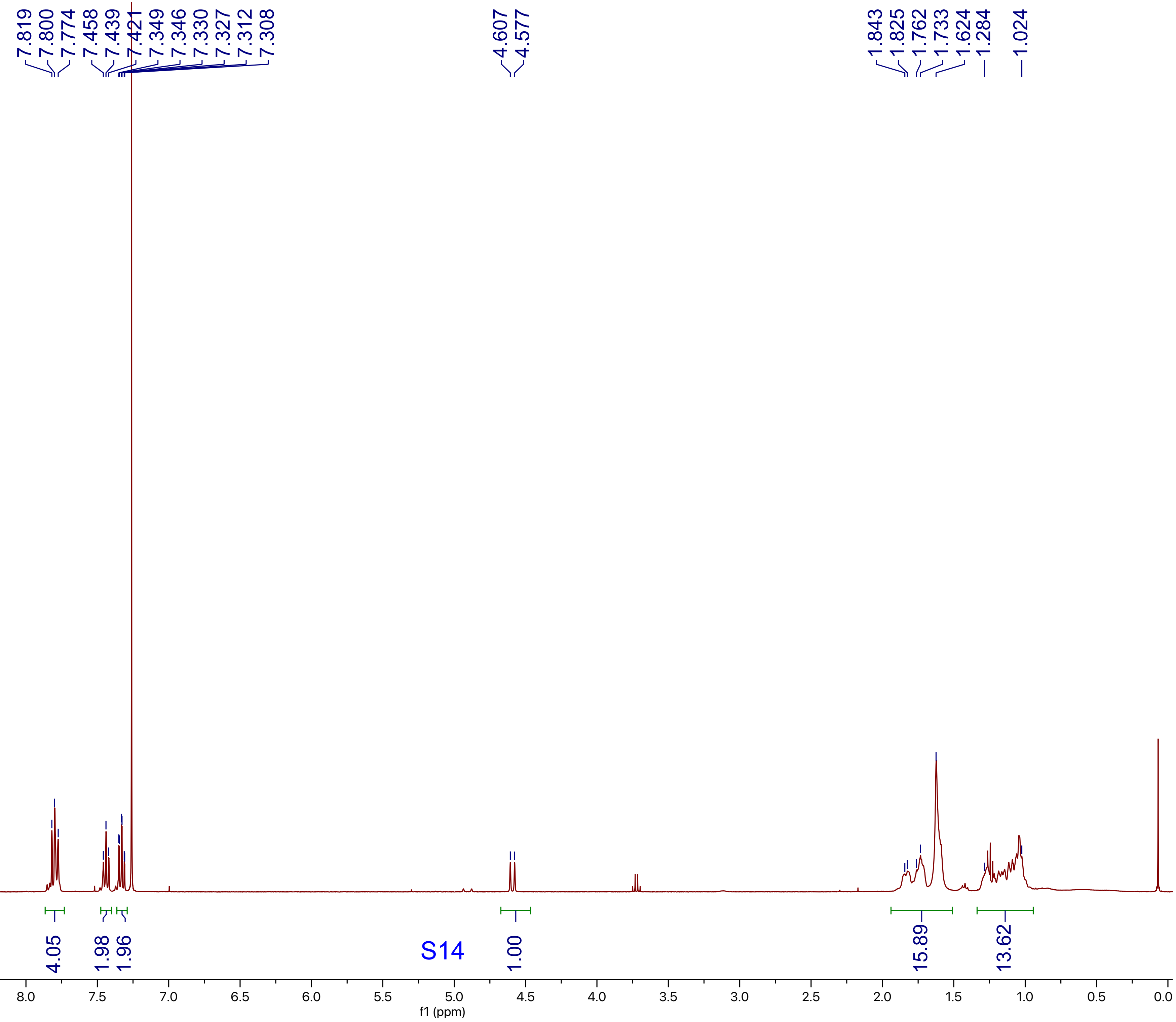

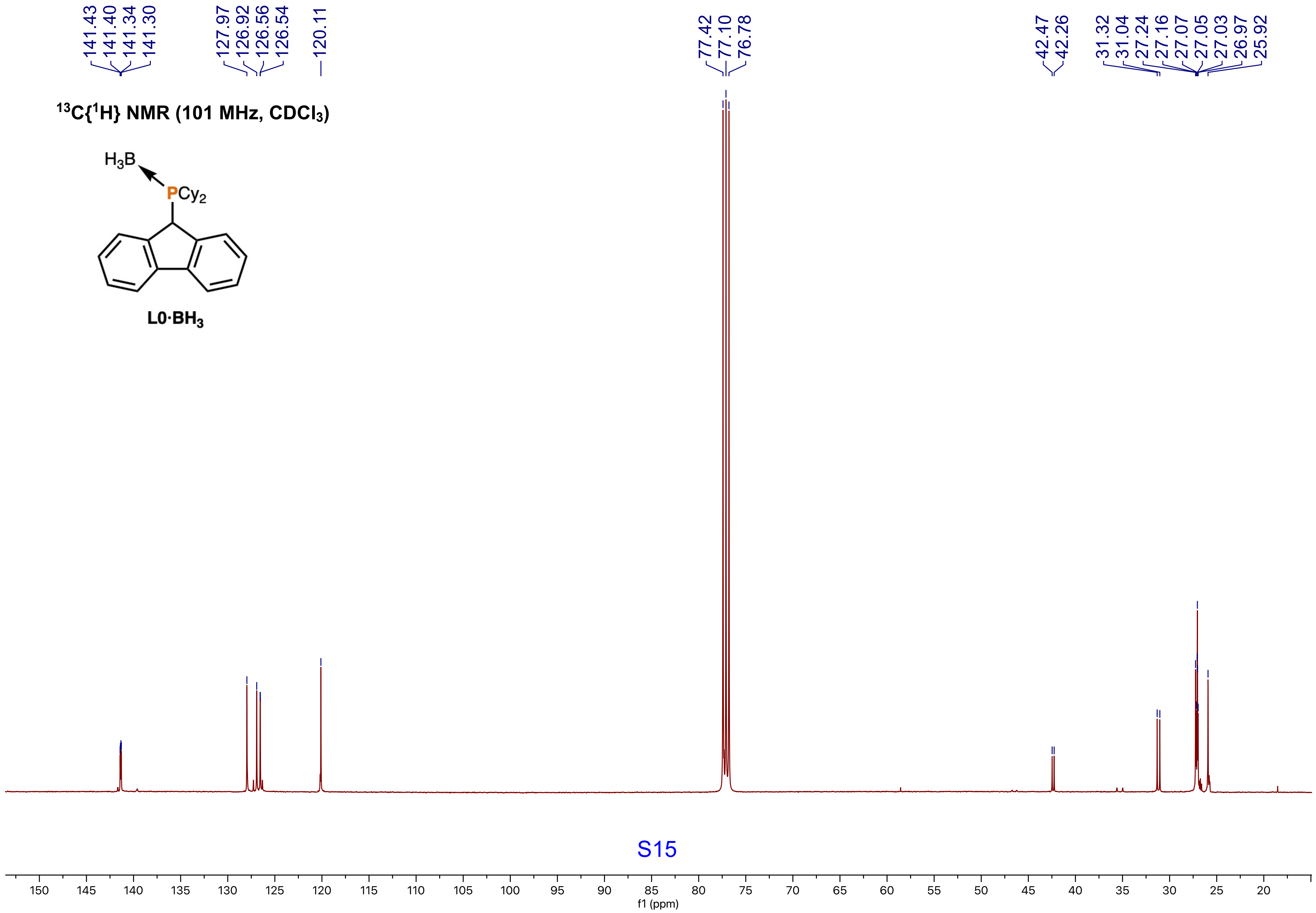
141.43
141.40
141.34
141.30
127.97
126.92
126.56
126.54
120.11
77.42
77.10
76.78
42.47
42.26
31.32
31.04
27.24
27.16
27.07
27.05
27.03
26.97
25.92
$^{13}C\{^{1}H\}$ NMR (101 MHz, $CDCl_3$)
H_3B
PCy$_2$
L0·BH$_3$
150
145
140
135
130
125
120
115
110
105
100
95
90
85
80
75
70
65
60
55
50
45
40
35
30
25
20
f1 (ppm)

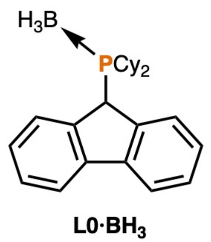

^{1}H-^{1}H COSY NMR ($CDCl_3$)

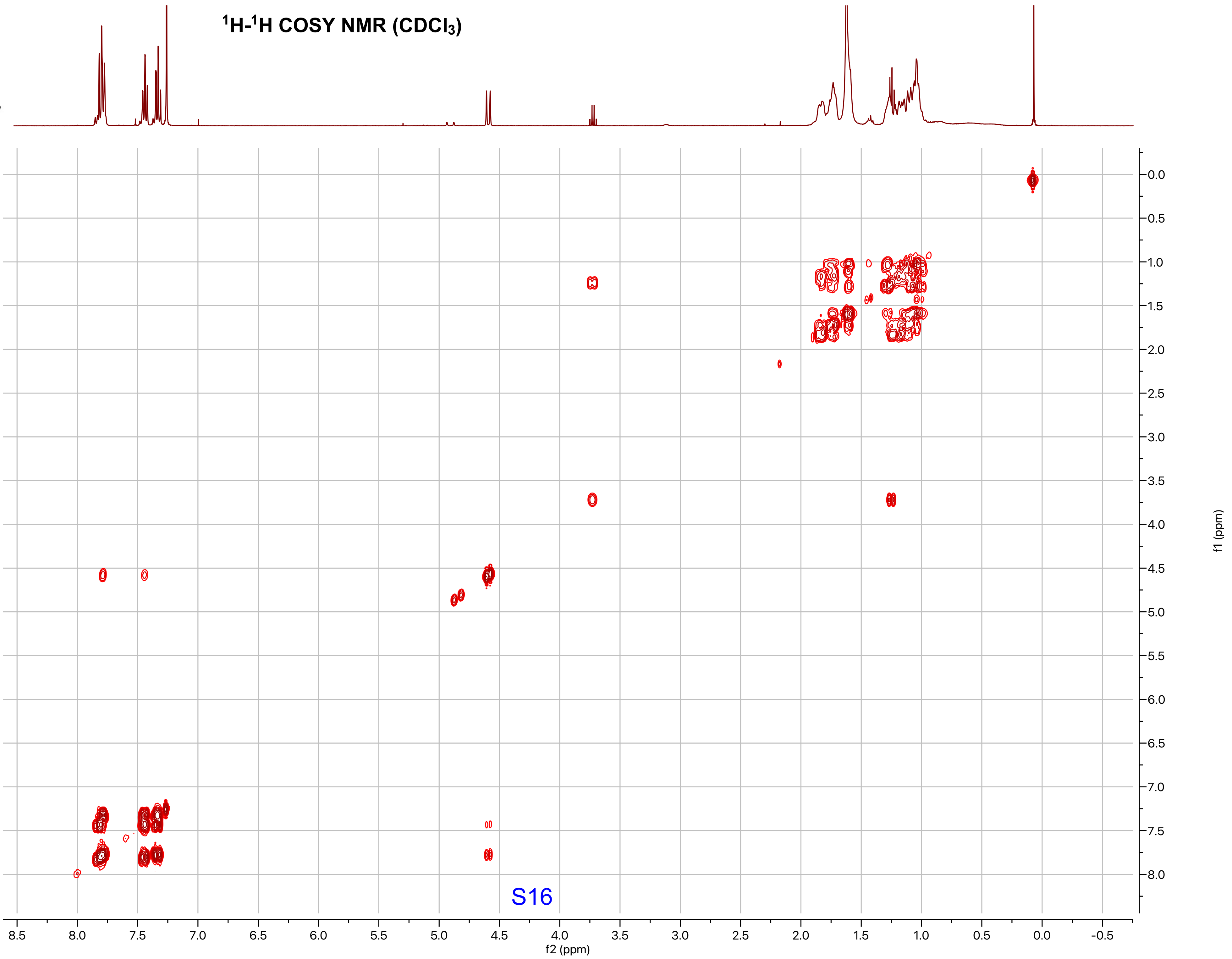

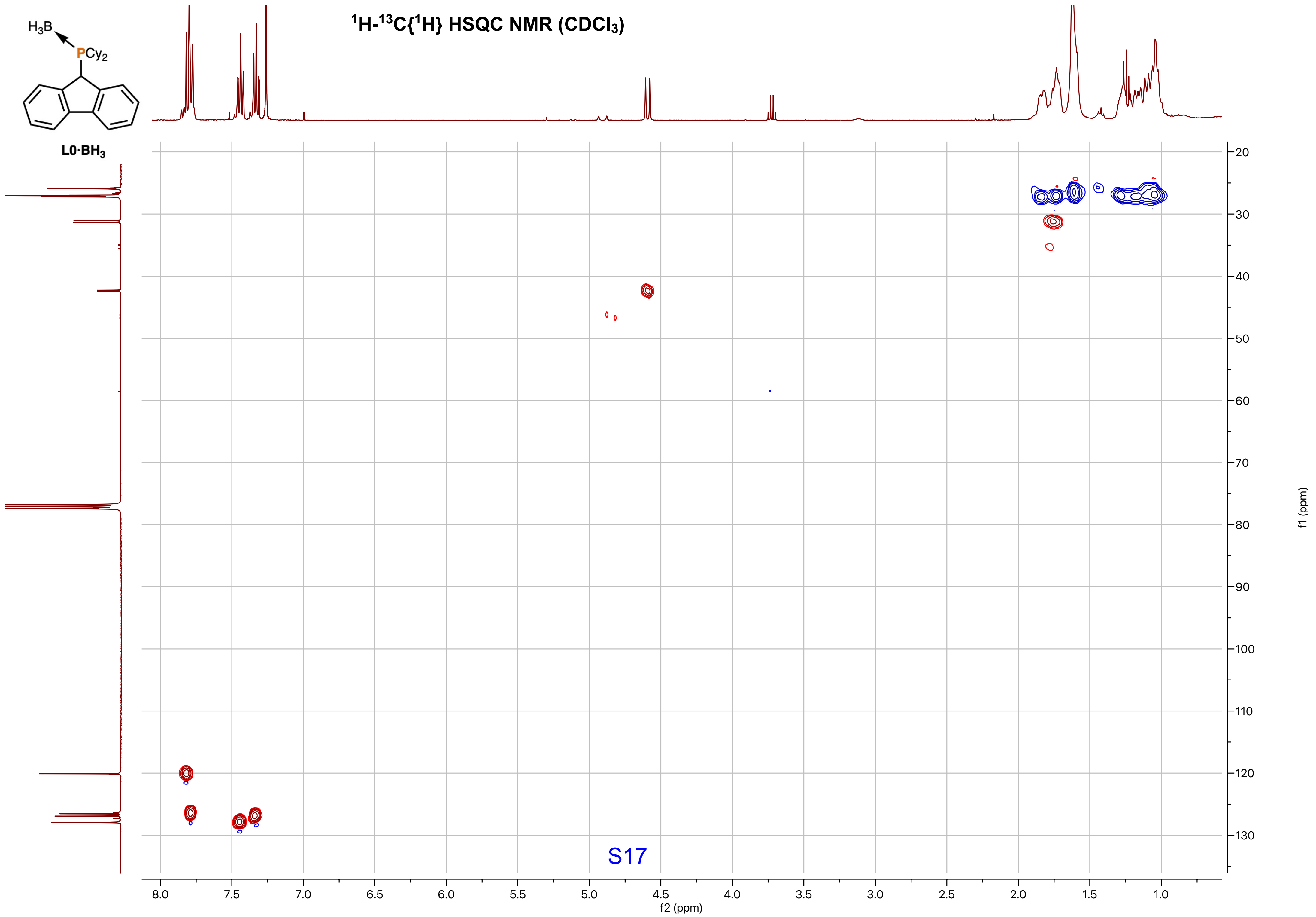

^1H-$^{13}C\{^1H\}$ HSQC NMR ($CDCl_3$)
H_3B
PCy$_2$
L0·BH$_3$
f2 (ppm)
f1 (ppm)

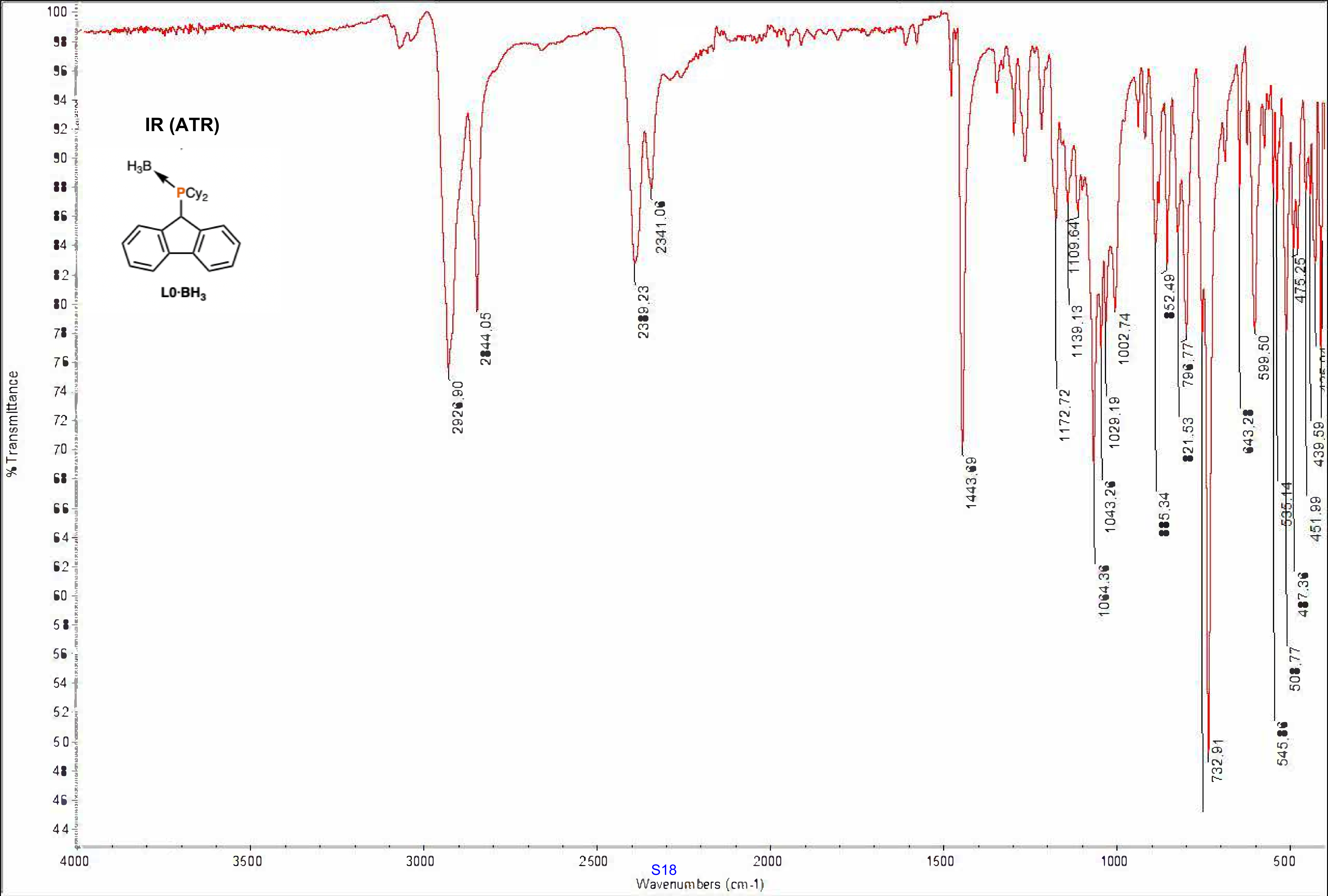
IR (ATR)
H3B
PCy2
L0·BH3
% Transmittance
Wavenumbers (cm-1)
2926.90
2844.05
2389.23
2341.06
1443.69
1172.72
1139.13
1109.64
1064.36
1043.26
1029.19
1002.74
885.34
852.49
821.53
796.77
732.91
643.28
599.50
545.86
535.14
508.77
487.36
475.25
451.99
439.59

HRMS

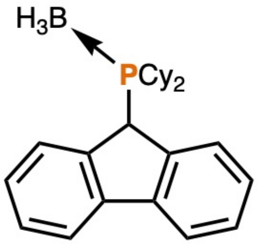

H_3B
PCy2

L0·BH$_3$

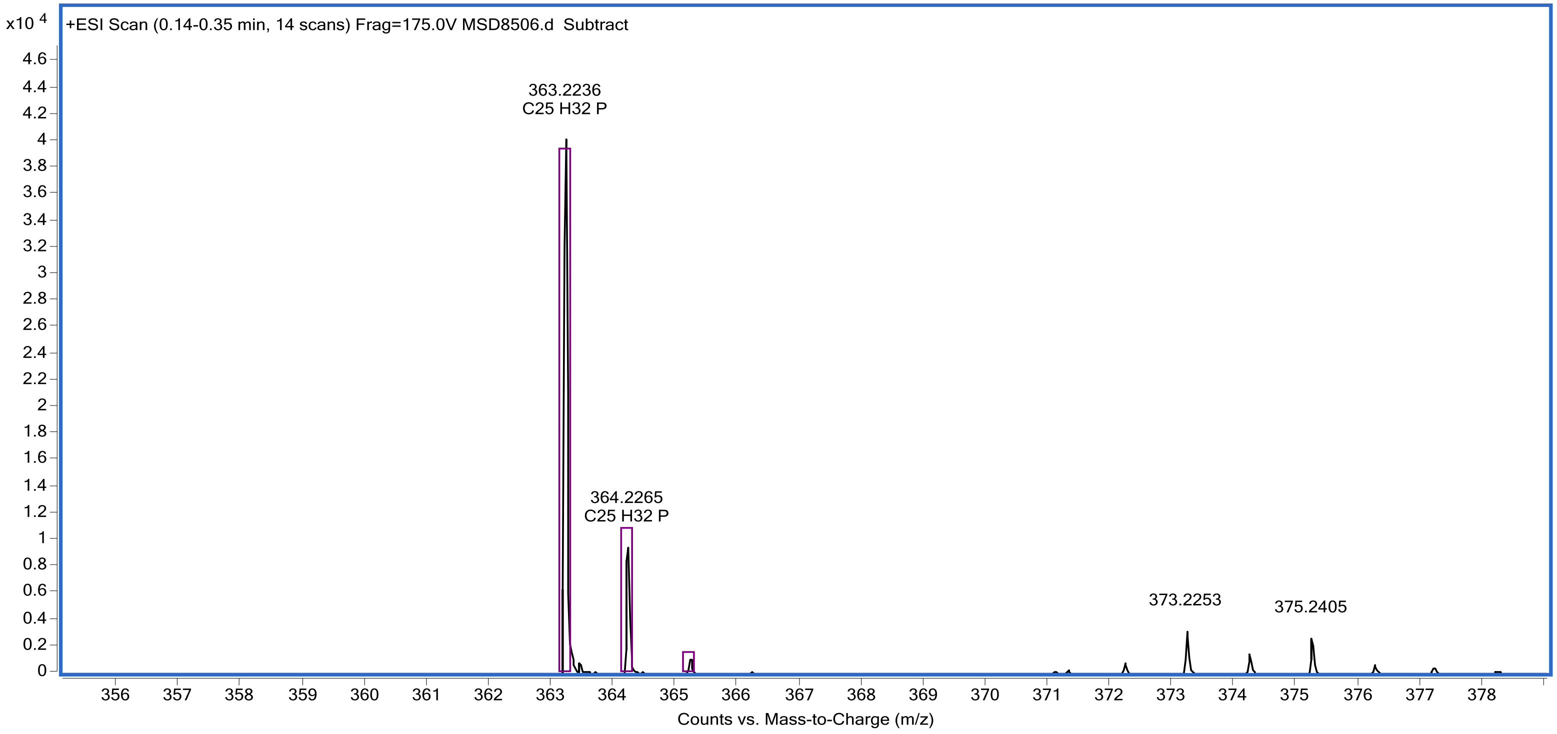

x10 4
+ESI Scan (0.14-0.35 min, 14 scans) Frag=175.0V MSD8506.d Subtract
363.2236
C25 H32 P
364.2265
C25 H32 P
373.2253
375.2405
Counts vs. Mass-to-Charge (m/z)

$^{31}P\{^{1}H\}$ NMR (162 MHz, C_6D_6)

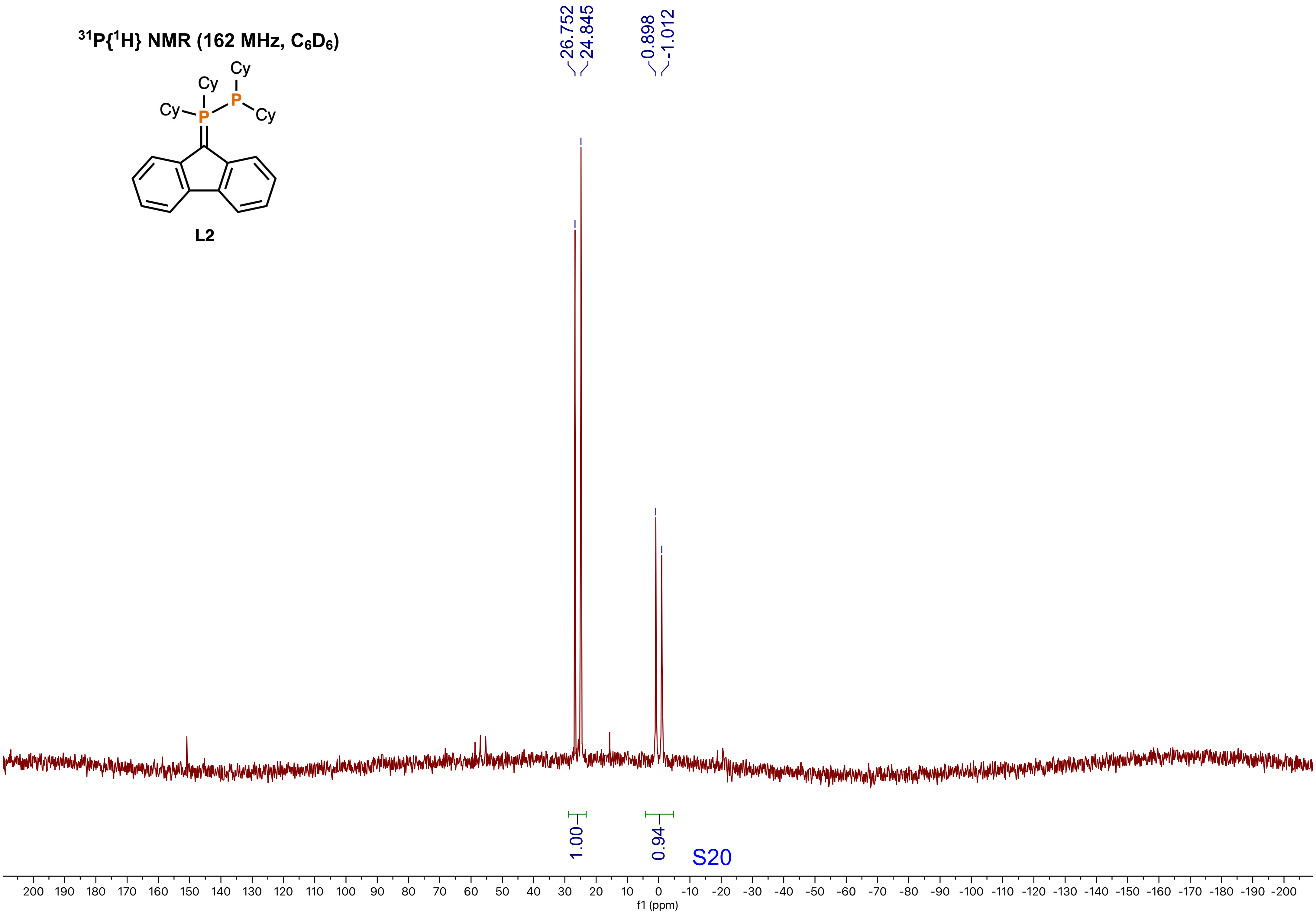

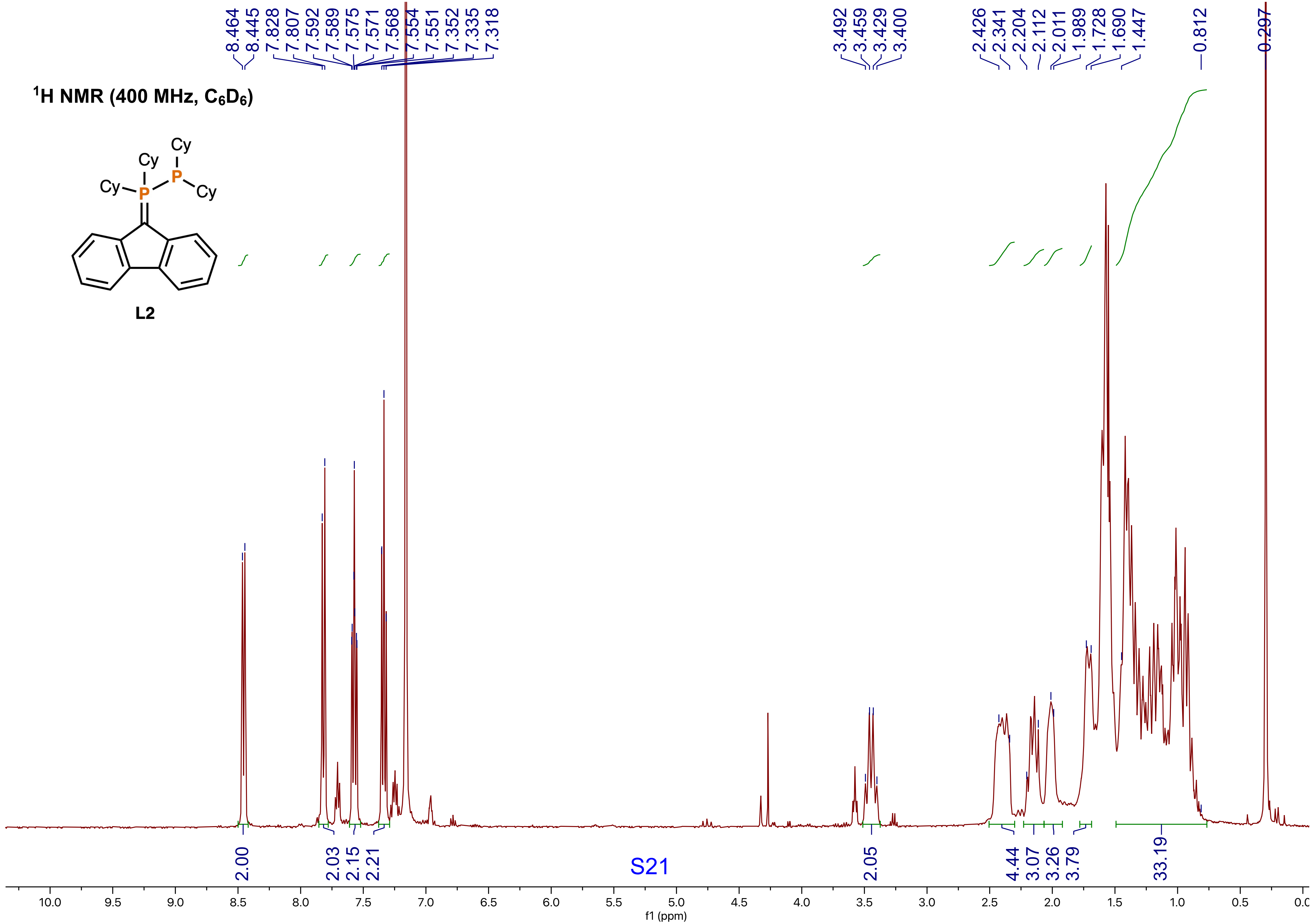

^{1}H NMR (400 MHz, C_6D_6)
Cy
Cy
P
P
Cy
Cy
L2
8.464
8.445
7.828
7.807
7.592
7.589
7.575
7.571
7.568
7.554
7.551
7.352
7.335
7.318
3.492
3.459
3.429
3.400
2.426
2.341
2.204
2.112
2.011
1.989
1.728
1.690
1.447
0.812
0.297
2.00
2.03
2.15
2.21
2.05
4.44
3.07
3.26
3.79
33.19
10.0
9.5
9.0
8.5
8.0
7.5
7.0
6.5
6.0
5.5
5.0
4.5
4.0
3.5
3.0
2.5
2.0
1.5
1.0
0.5
f1 (ppm)

^{1}H NMR (400 MHz, $CDCl_3$)

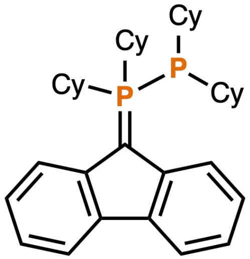

L2

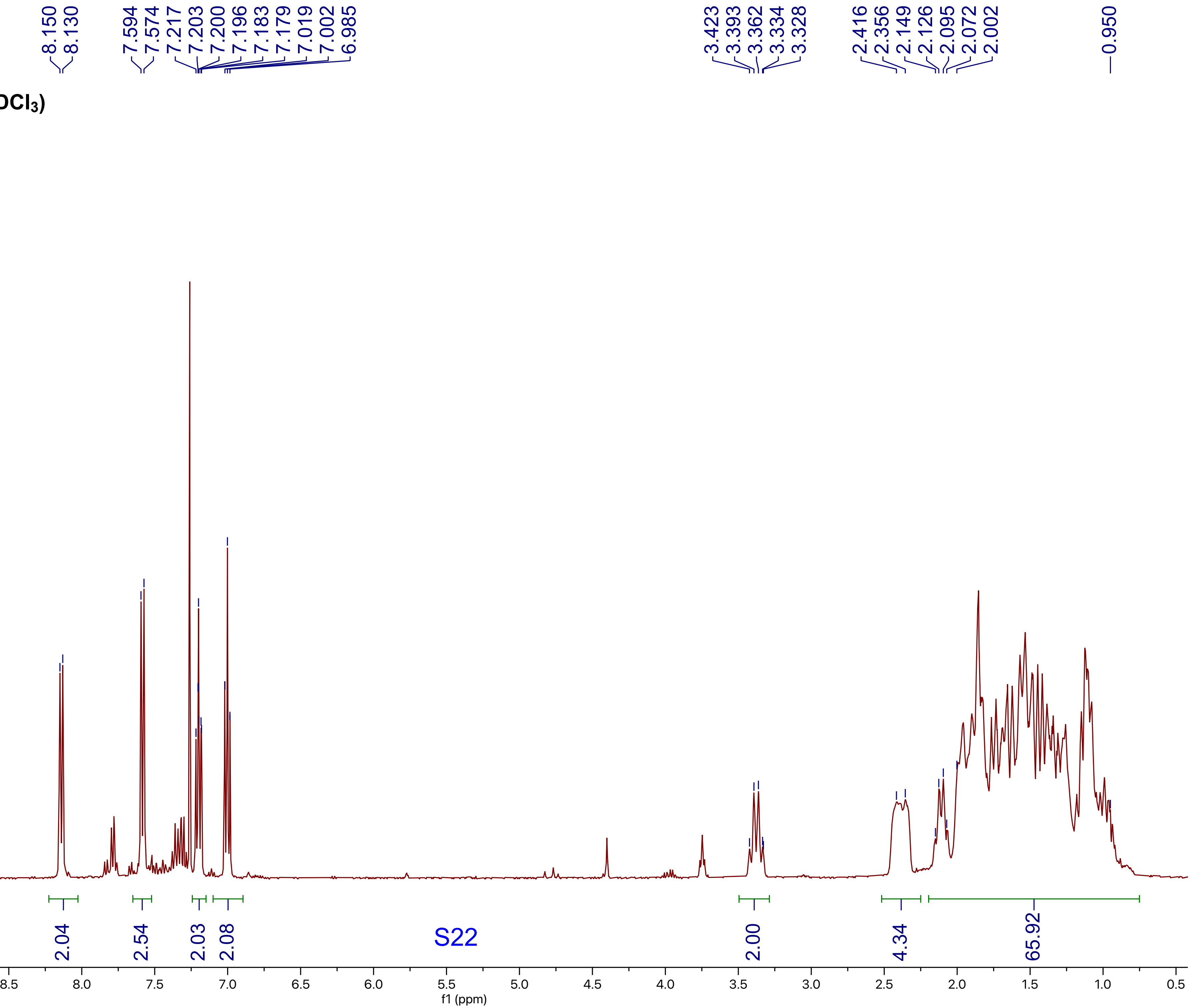

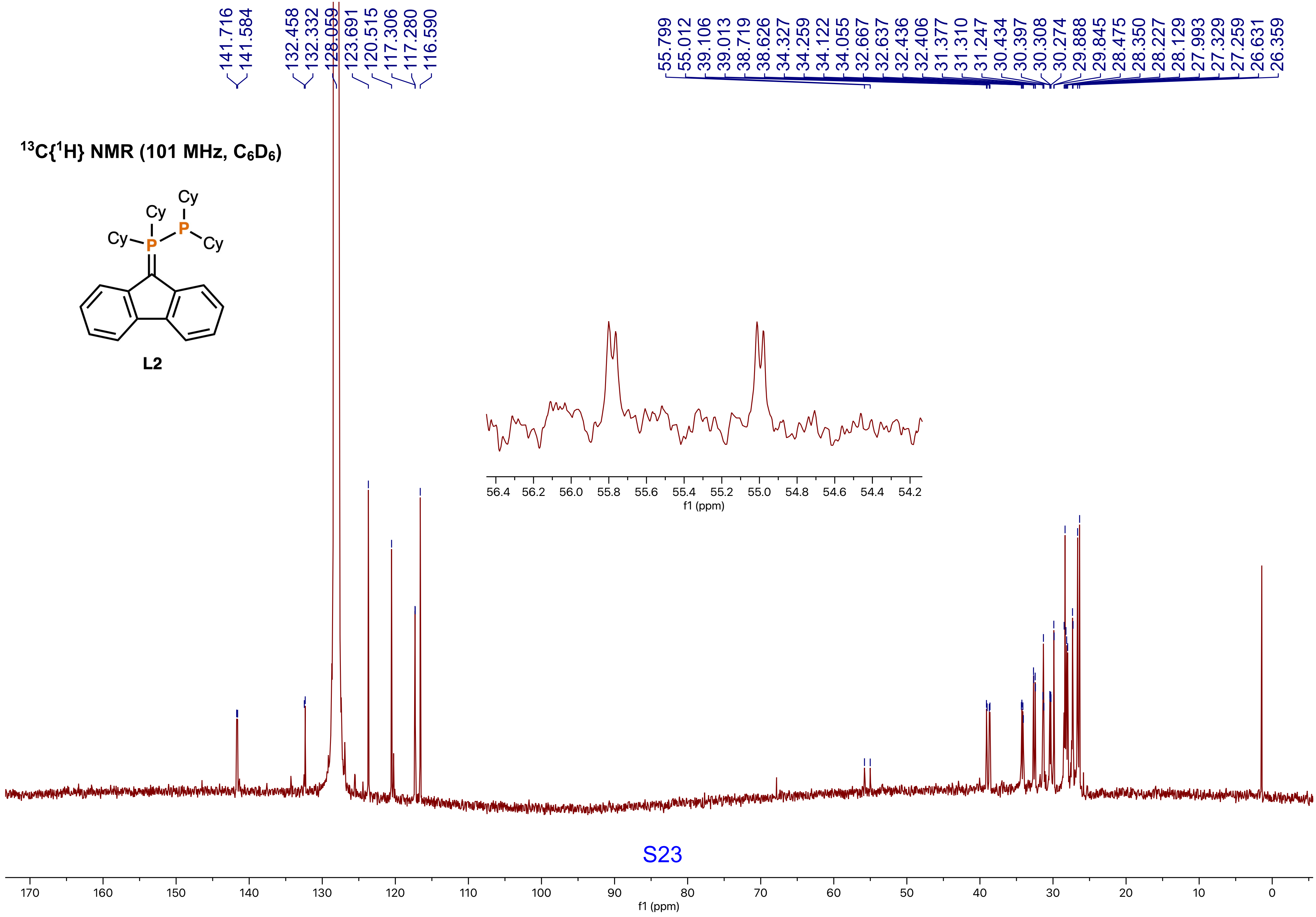

141.716
141.584
132.458
132.332
128.059
123.691
120.515
117.306
117.280
116.590
55.799
55.012
39.106
39.013
38.719
38.626
34.327
34.259
34.122
34.055
32.667
32.637
32.436
32.406
31.377
31.310
31.247
30.434
30.397
30.308
30.274
29.888
29.845
28.475
28.350
28.227
28.129
27.993
27.329
27.259
26.631
26.359
$^{13}C\{^1H\}$ NMR (101 MHz, C_6D_6)
Cy
P
L2
56.4 56.2 56.0 55.8 55.6 55.4 55.2 55.0 54.8 54.6 54.4 54.2
f1 (ppm)
170 160 150 140 130 120 110 100 90 80 70 60 50 40 30 20 10 0
f1 (ppm)

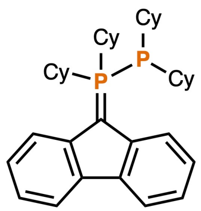

L2

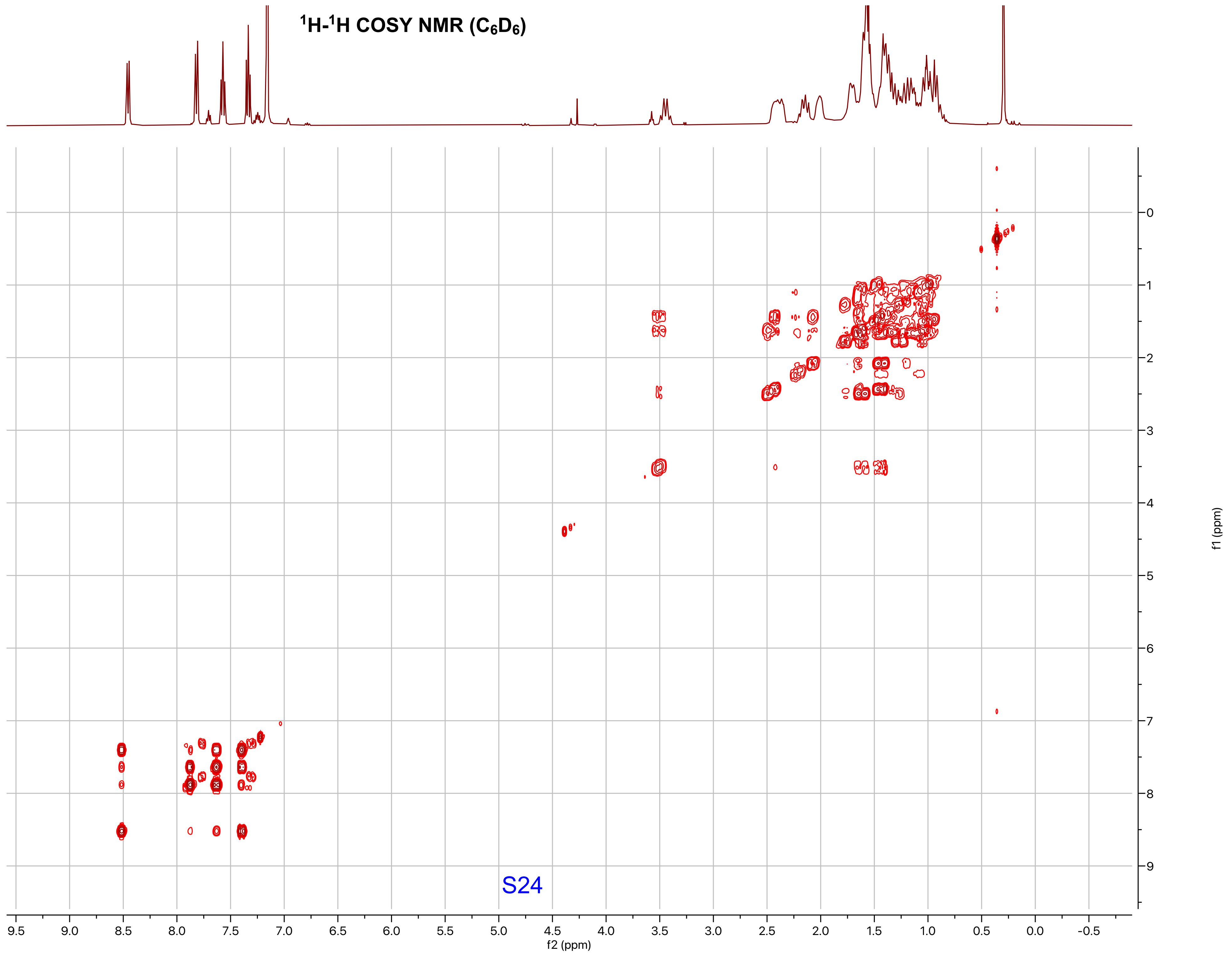

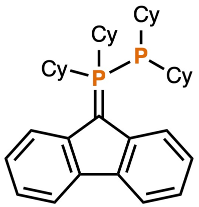

L2

^{1}H-^{13}C{^{1}H} HSQC NMR (C_6D_6)

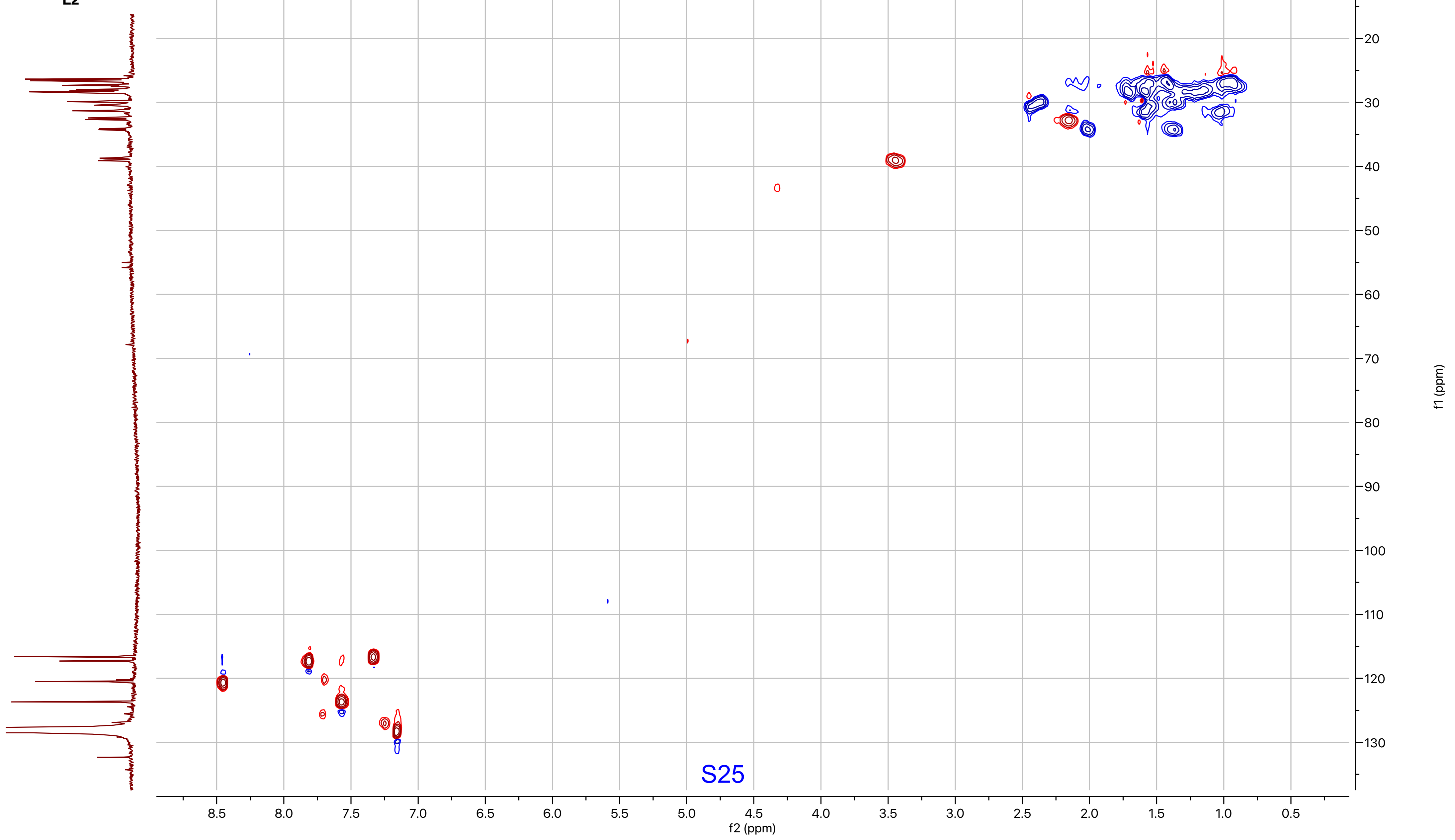

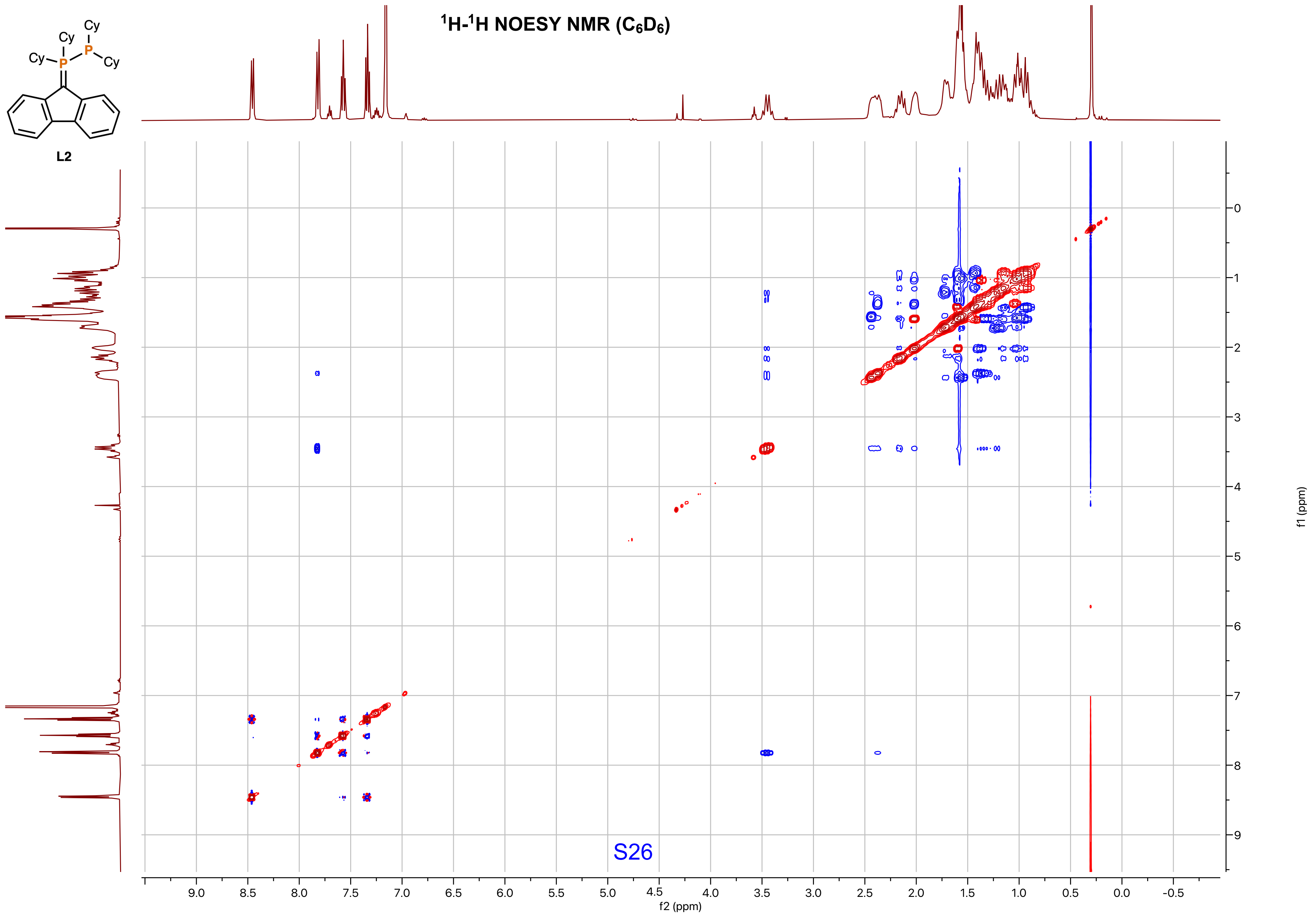
Cy
Cy
Cy
P
P
Cy
L2
^{1}H-^{1}H NOESY NMR (C_6D_6)
f2 (ppm)
f1 (ppm)

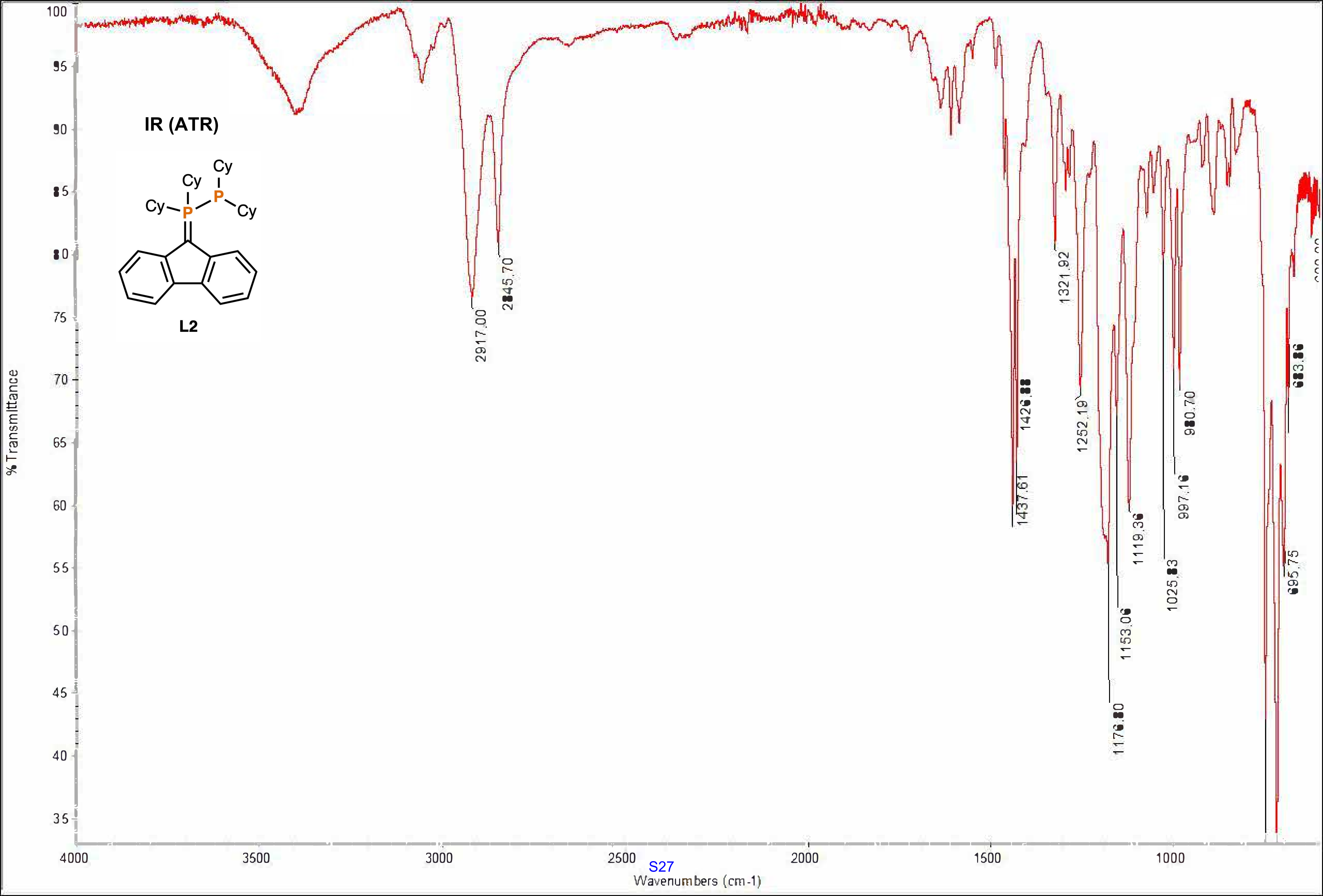
IR (ATR)
Cy
P
L2
% Transmittance
Wavenumbers (cm-1)
2917.00
1437.61
1321.92
1252.19
980.70
695.75

HRMS

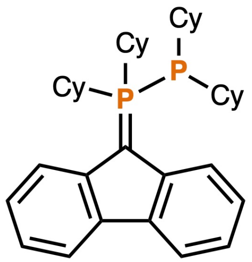
Cy
Cy
P
P
Cy
Cy

L2

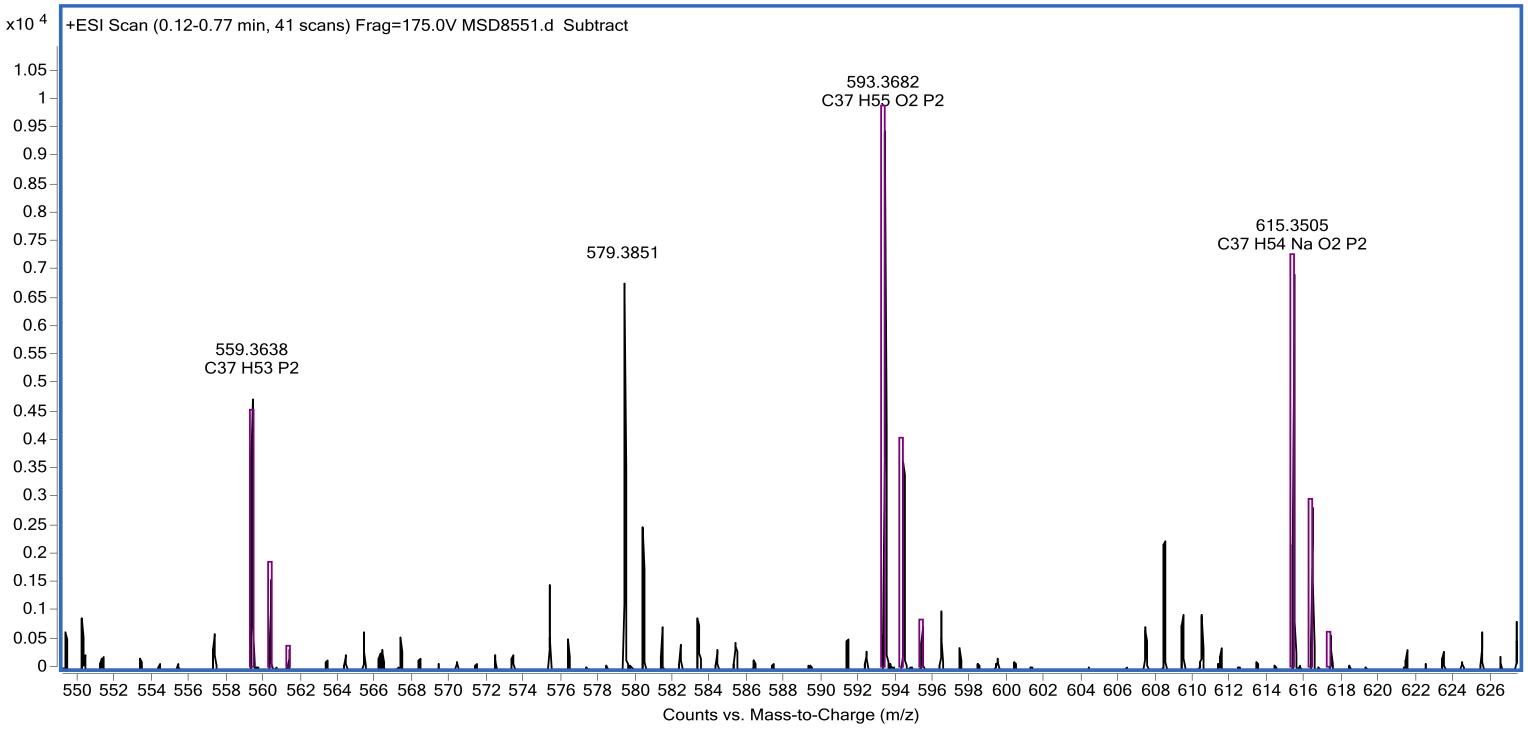
x10 4
+ESI Scan (0.12-0.77 min, 41 scans) Frag=175.0V MSD8551.d Subtract
593.3682
C37 H55 O2 P2
615.3505
C37 H54 Na O2 P2
579.3851
559.3638
C37 H53 P2
Counts vs. Mass-to-Charge (m/z)

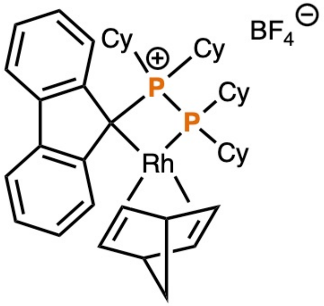

C1

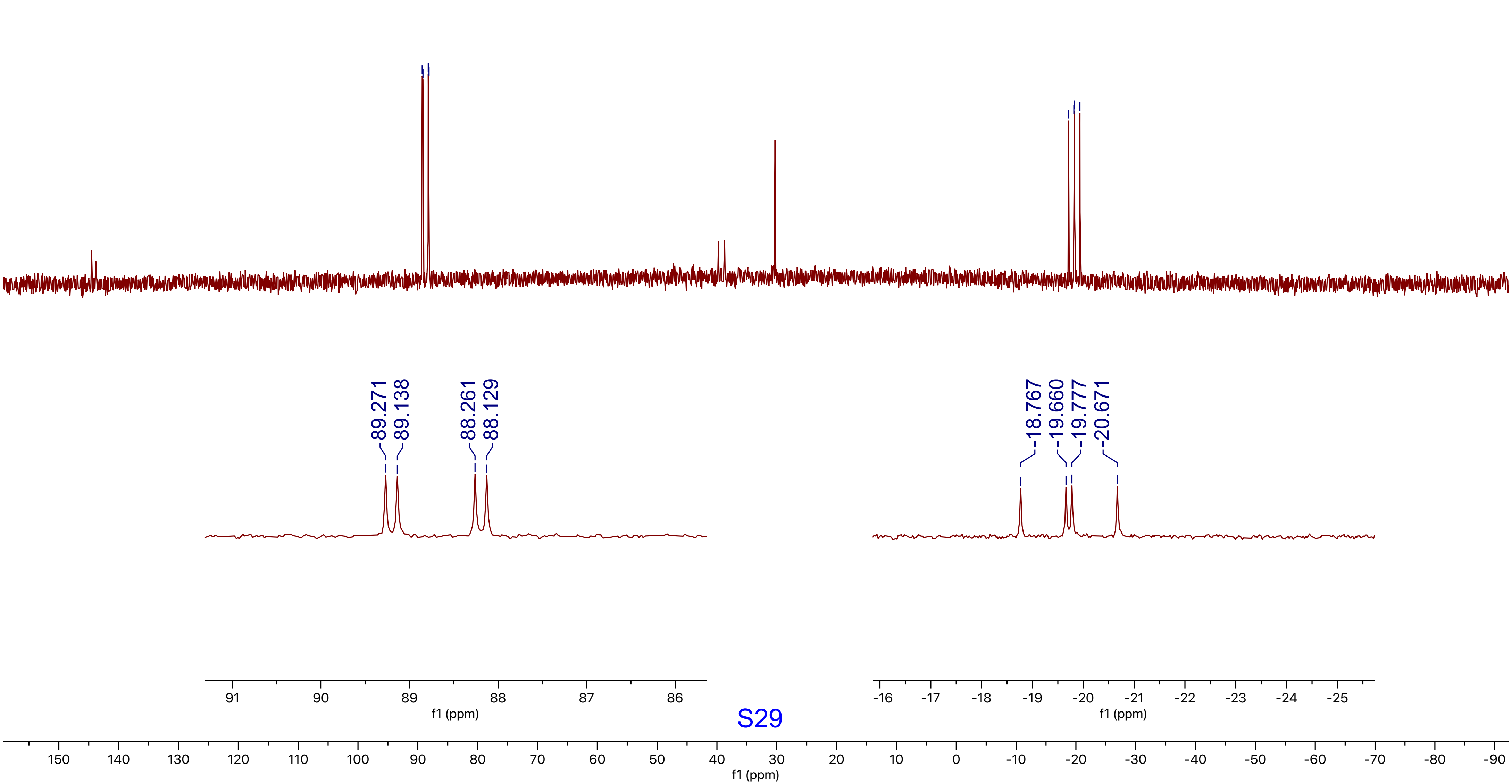

$^{31}P\{^1H\}$ NMR (162 MHz, CD_2Cl_2)
89.271
89.138
88.261
88.129
-18.767
-19.660
-19.777
-20.671
f1 (ppm)

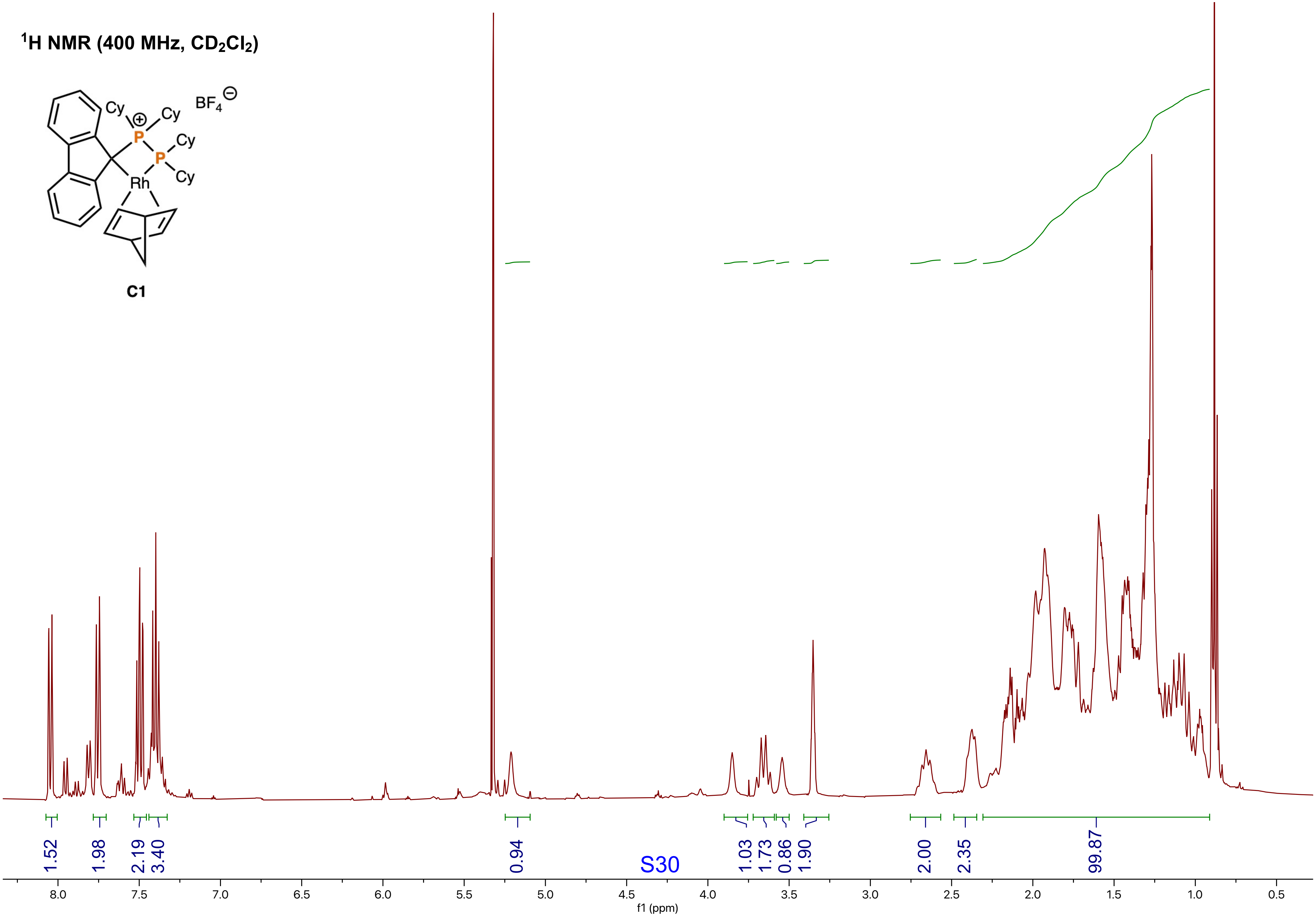

1H NMR (400 MHz, CD2Cl2)
Cy
Cy
Cy
Cy
P
P
Rh
BF4
C1
1.52
1.98
2.19
3.40
0.94
1.03
1.73
0.86
1.90
2.00
2.35
99.87
8.0
7.5
7.0
6.5
6.0
5.5
5.0
4.5
4.0
3.5
3.0
2.5
2.0
1.5
1.0
0.5
f1 (ppm)

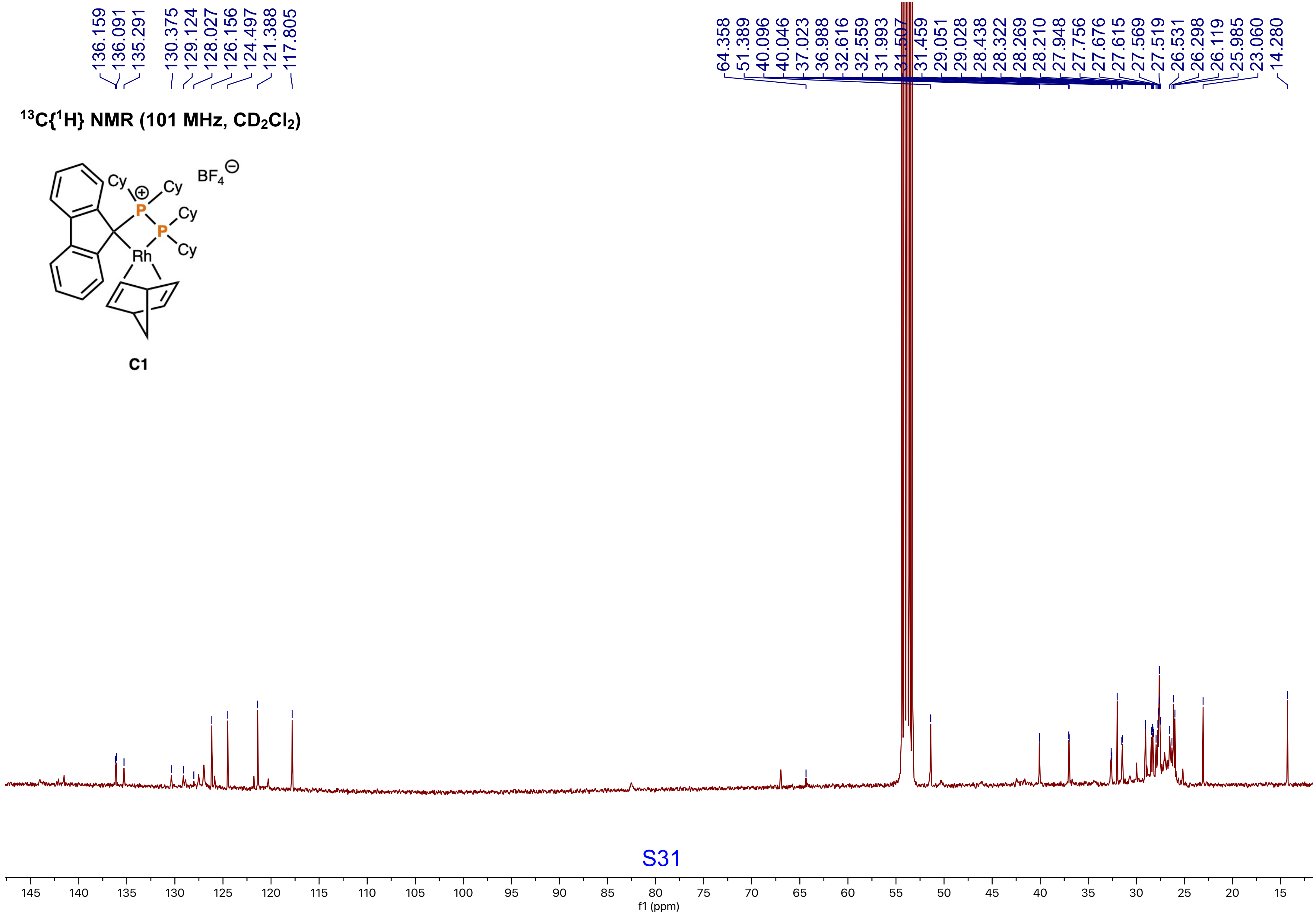
136.159
136.091
135.291
130.375
129.124
128.027
126.156
124.497
121.388
117.805
64.358
51.389
40.096
40.046
37.023
36.988
32.616
32.559
31.993
31.507
31.459
29.051
29.028
28.438
28.322
28.269
28.210
27.948
27.756
27.676
27.615
27.569
27.519
26.531
26.298
26.119
25.985
23.060
14.280
$^{13}C\{^1H\}$ NMR (101 MHz, CD_2Cl_2)
Cy
Cy
Cy
Cy
P
P
Rh
BF_4
C1
145
140
135
130
125
120
115
110
105
100
95
90
85
80
75
70
65
60
55
50
45
40
35
30
25
20
15
f1 (ppm)

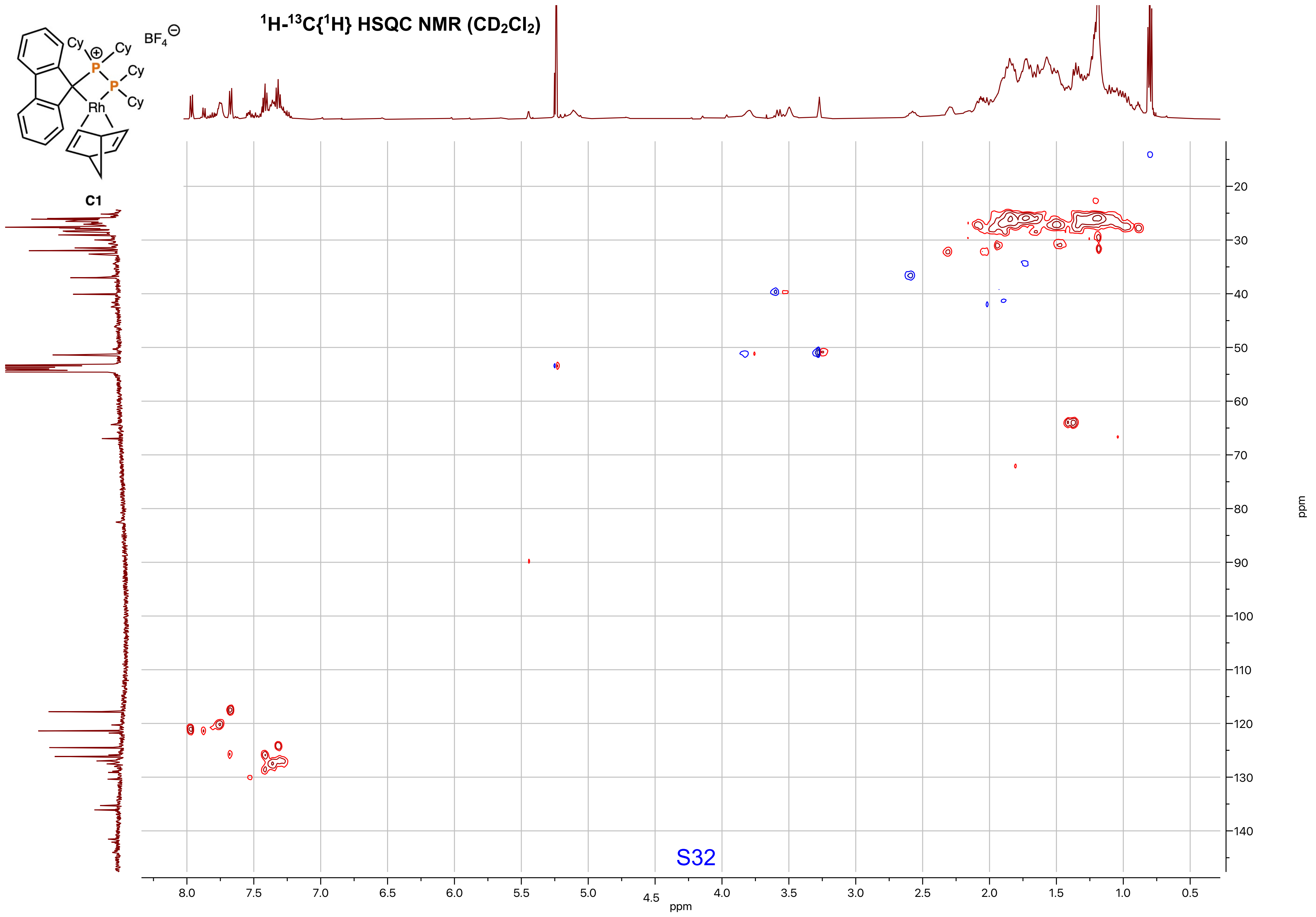
^1H-$^{13}C\{^1H\}$ HSQC NMR (CD_2Cl_2)
Cy
Cy
Cy
Cy
P
P
Rh
$BF_4^{\ominus}$
C1
ppm
ppm

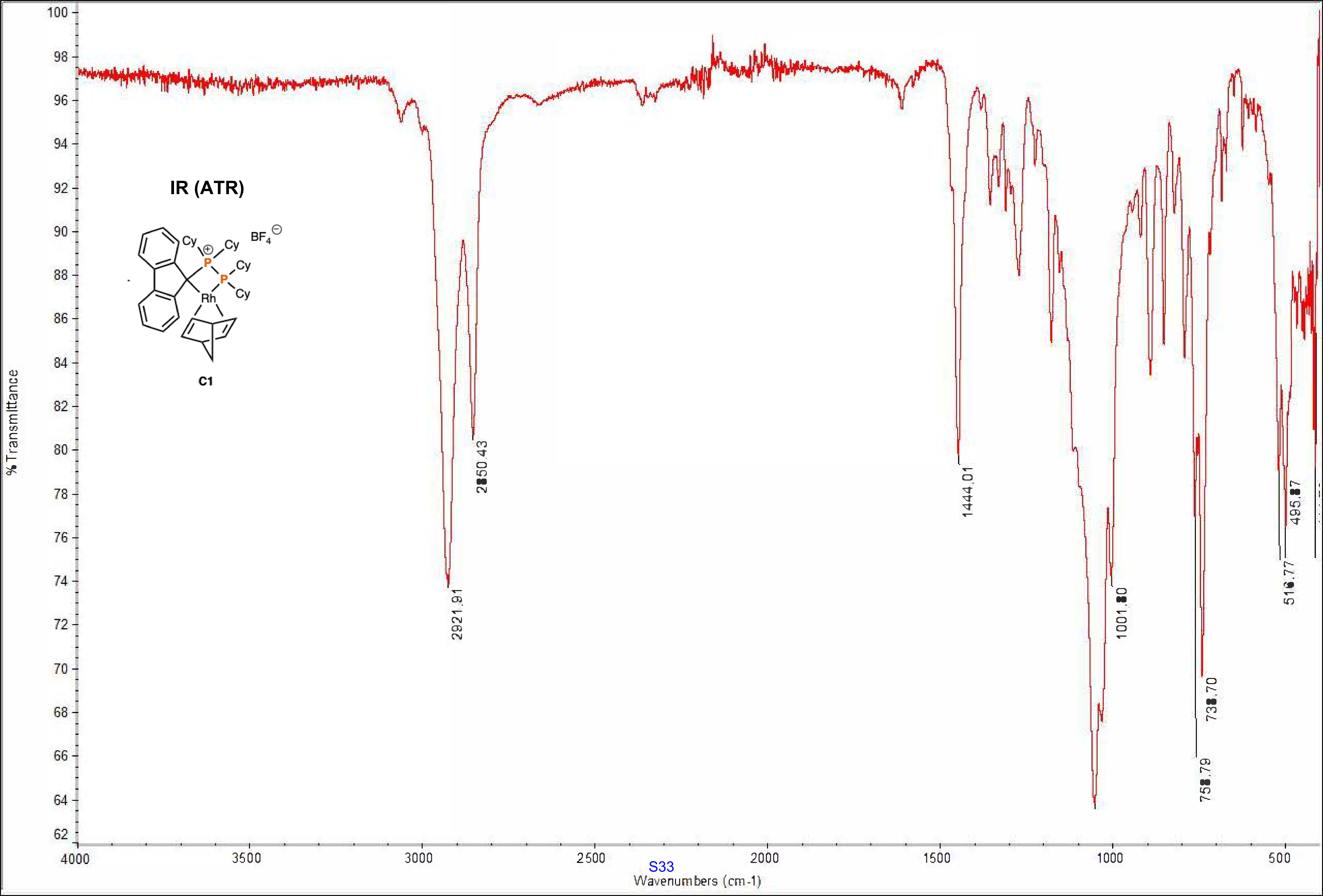

IR (ATR)
Cy
Cy
Cy
Cy
P
P
Rh
$BF_4^{\ominus}$
C1
% Transmittance
Wavenumbers (cm-1)
2921.91
1444.01

HRMS

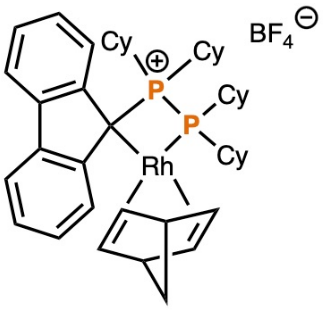
Cy
Cy
Cy
Cy
$BF_4^{\ominus}$
P
P
Rh

C1

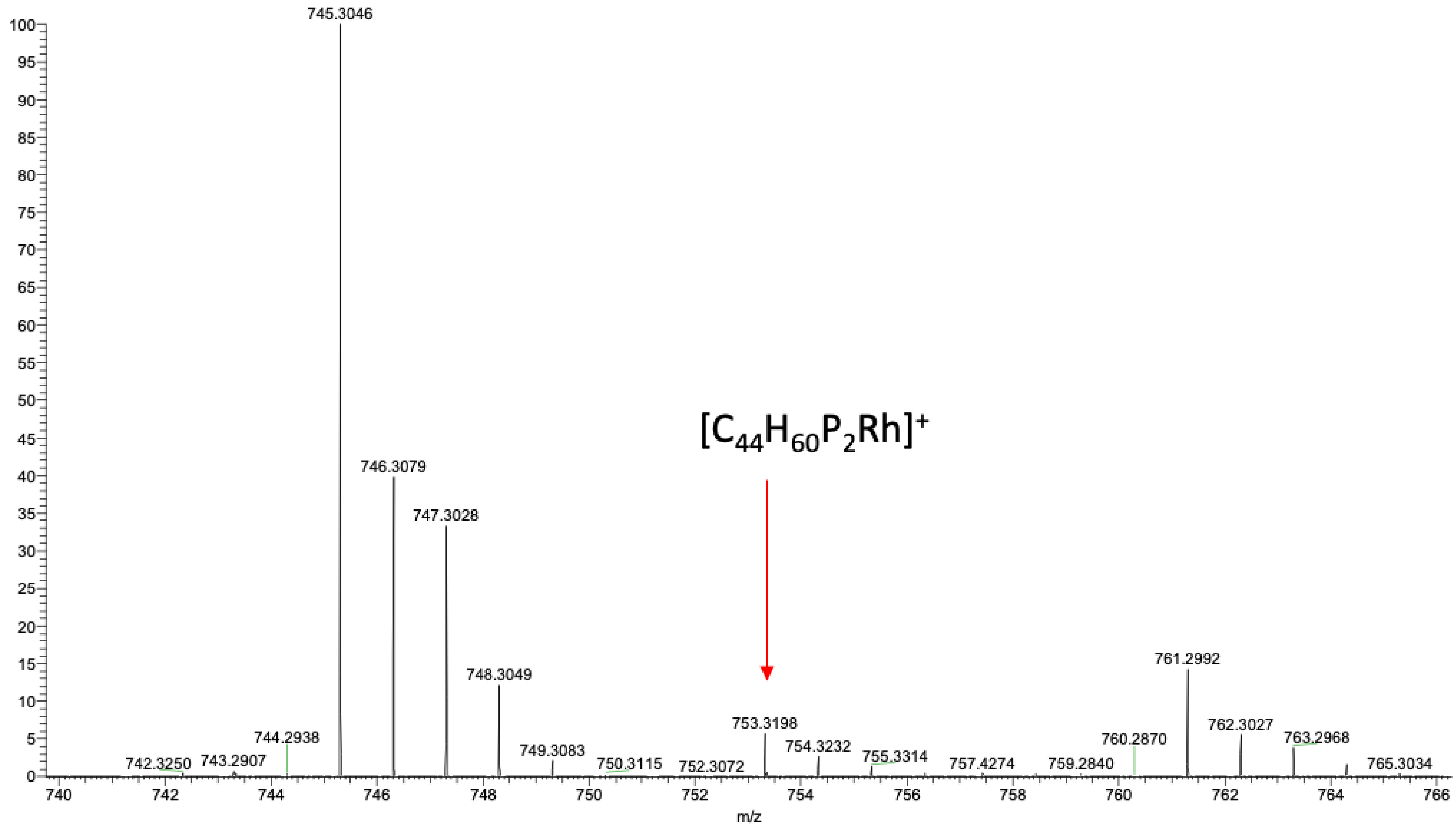
$[C_{44}H_{60}P_2Rh]^+$
745.3046
746.3079
747.3028
748.3049
749.3083
750.3115
752.3072
753.3198
754.3232
755.3314
757.4274
759.2840
760.2870
761.2992
762.3027
763.2968
765.3034
742.3250
743.2907
744.2938
m/z

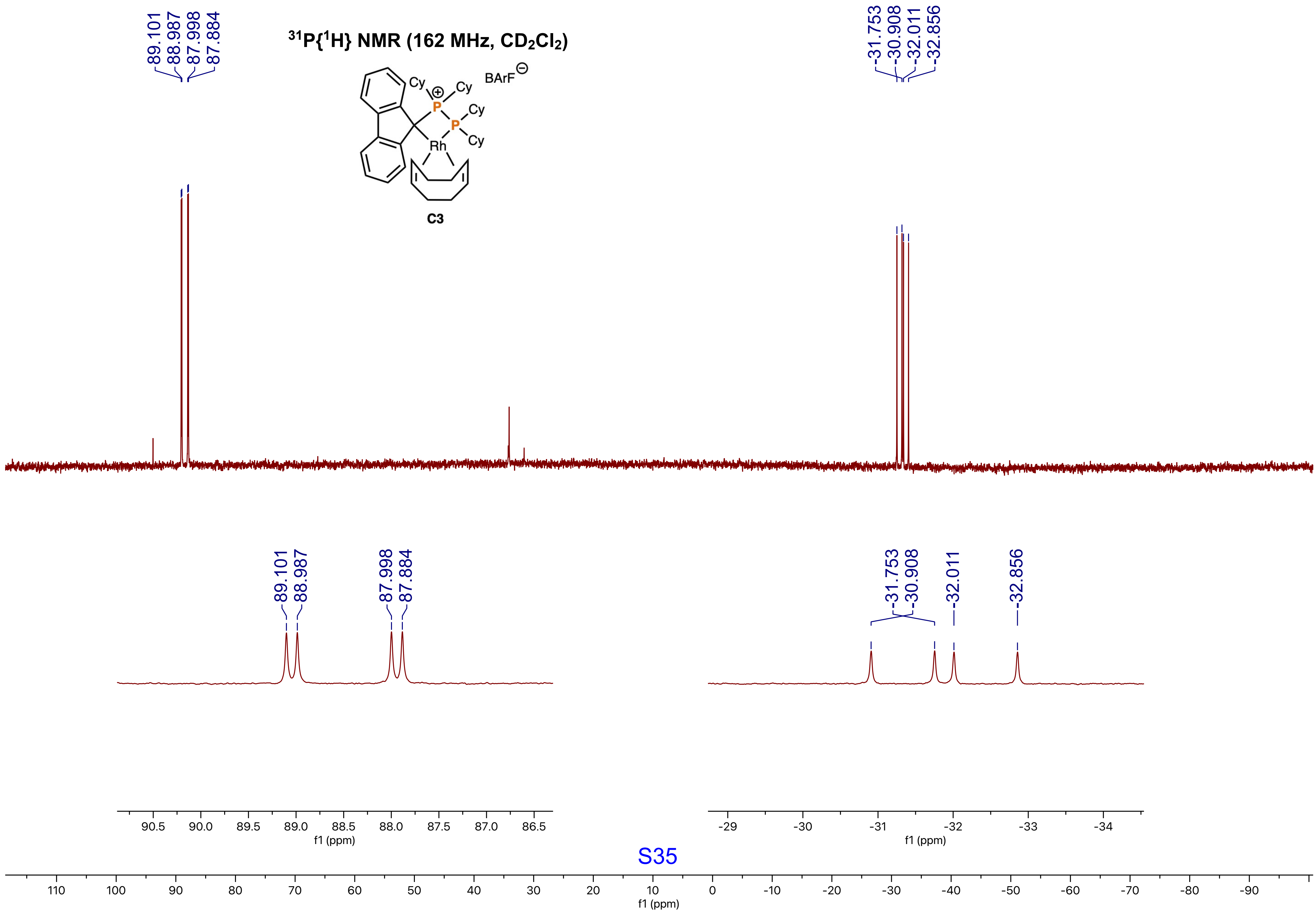

$^{31}P\{^{1}H\}$ NMR (162 MHz, CD_2Cl_2)
89.101
88.987
87.998
87.884
-31.753
-30.908
-32.011
-32.856
Cy
Cy
Cy
Cy
P
P
Rh
BArF
C3
f1 (ppm)
f1 (ppm)
f1 (ppm)

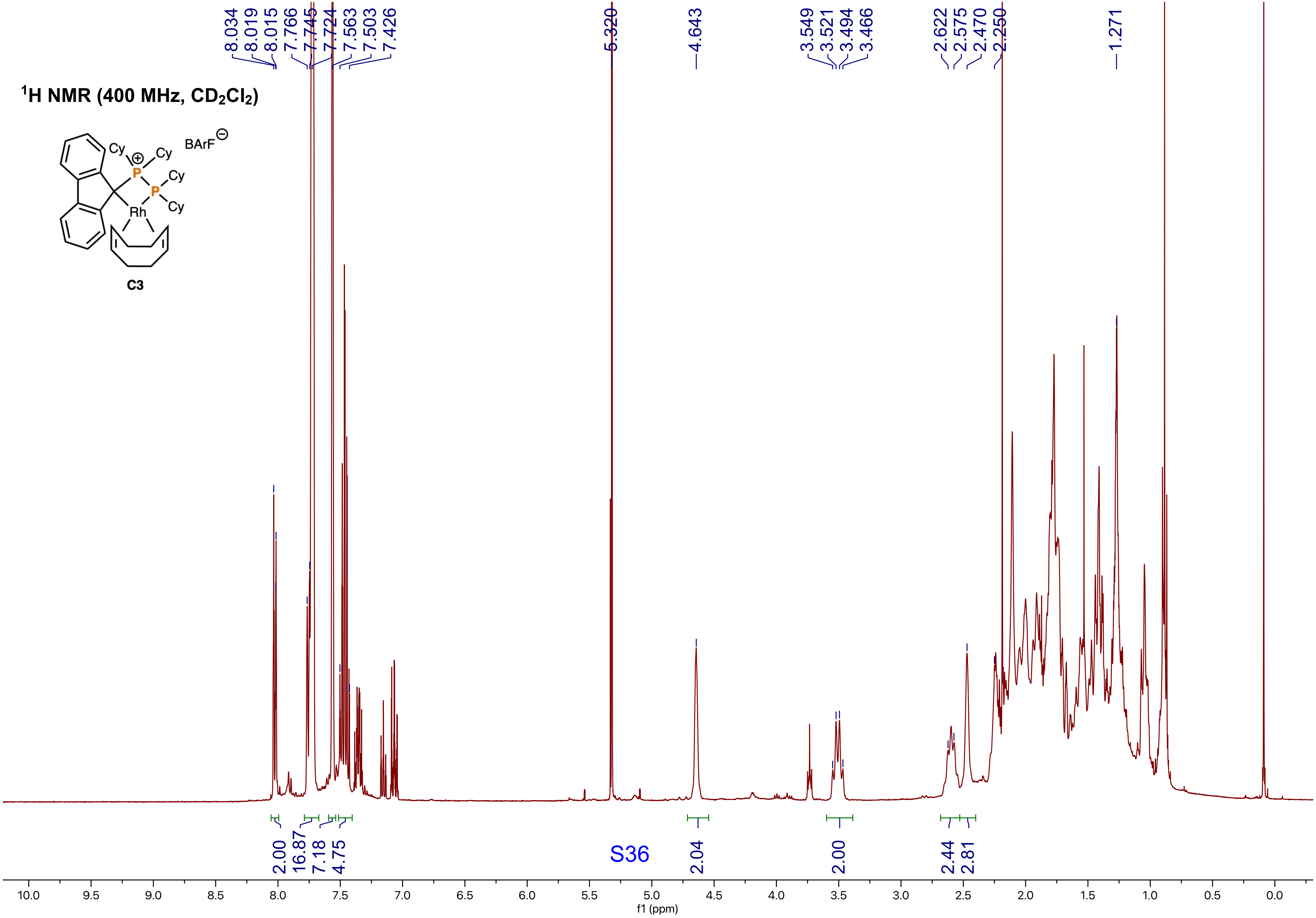
^{1}H NMR (400 MHz, CD_2Cl_2)
Cy
Cy
Cy
Cy
P
P
Rh
BArF
C3
8.034
8.019
8.015
7.766
7.745
7.724
7.563
7.503
7.426
5.320
4.643
3.549
3.521
3.494
3.466
2.622
2.575
2.470
2.250
1.271
2.00
16.87
7.18
4.75
2.04
2.00
2.44
2.81
10.0 9.5 9.0 8.5 8.0 7.5 7.0 6.5 6.0 5.5 5.0 4.5 4.0 3.5 3.0 2.5 2.0 1.5 1.0 0.5 0.0
f1 (ppm)

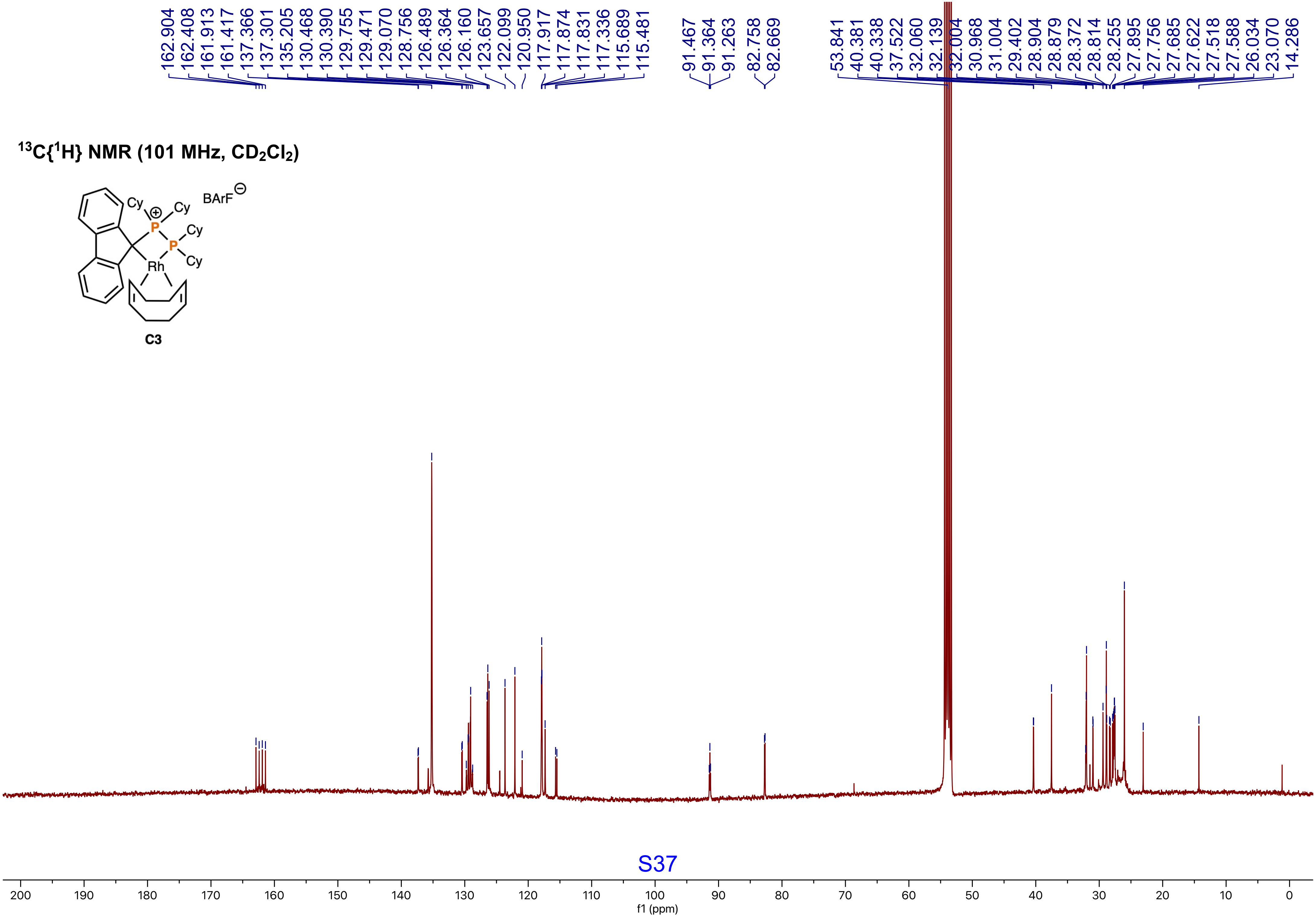

162.904
162.408
161.913
161.417
137.366
137.301
135.205
130.468
130.390
129.755
129.471
129.070
128.756
126.489
126.364
126.160
123.657
122.099
120.950
117.917
117.874
117.831
117.336
115.689
115.481
91.467
91.364
91.263
82.758
82.669
53.841
40.381
40.338
37.522
32.060
32.139
32.004
30.968
31.004
29.402
28.904
28.879
28.372
28.814
28.255
27.895
27.756
27.685
27.622
27.518
27.588
26.034
23.070
14.286
$^{13}C\{^1H\}$ NMR (101 MHz, CD_2Cl_2)
Cy
Cy
Cy
Cy
P
P
Rh
BArF
C3
200 190 180 170 160 150 140 130 120 110 100 90 80 70 60 50 40 30 20 10 0
f1 (ppm)

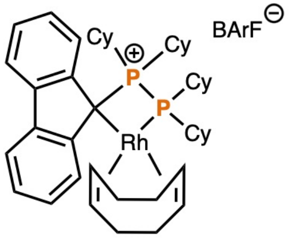

C3

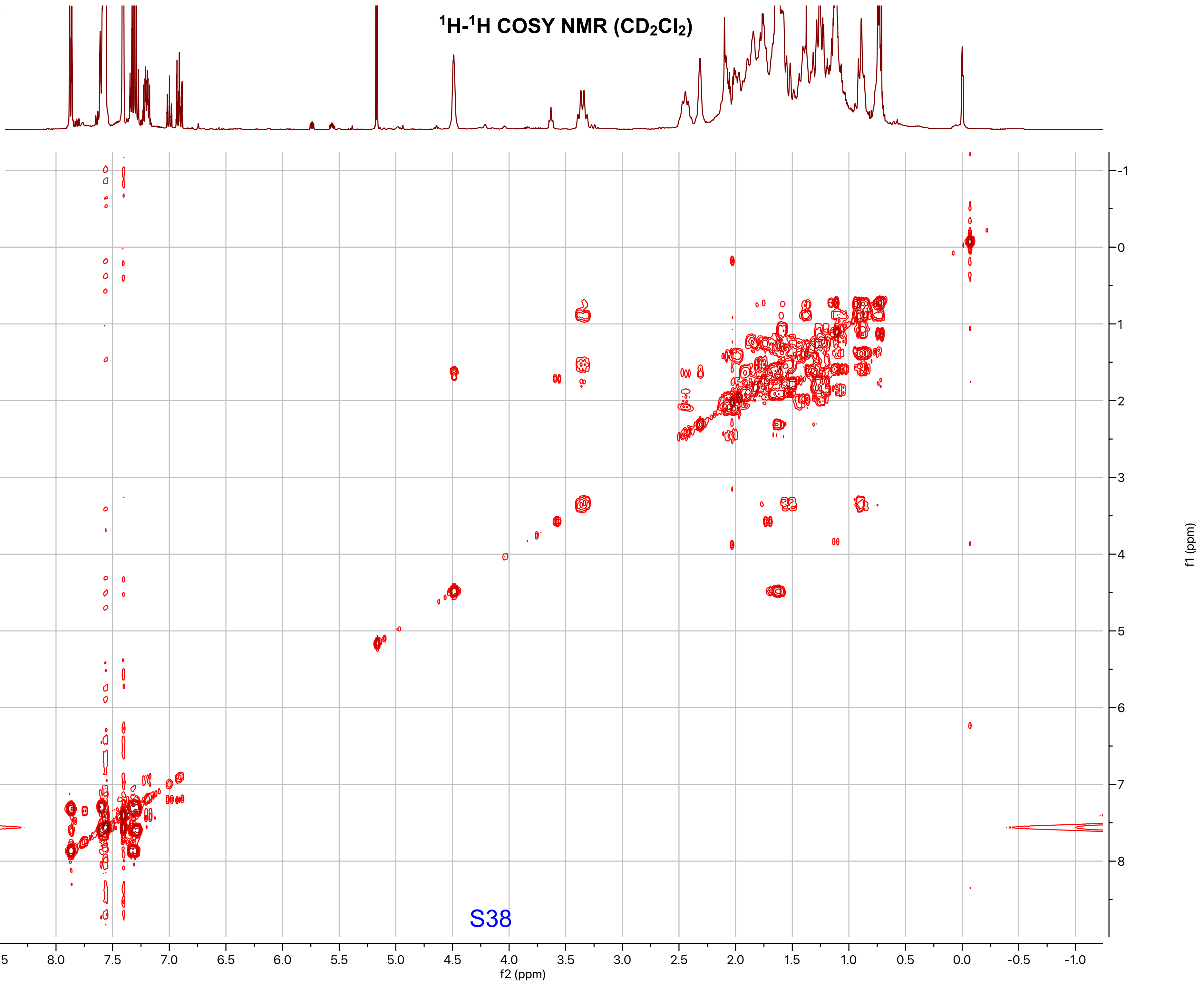

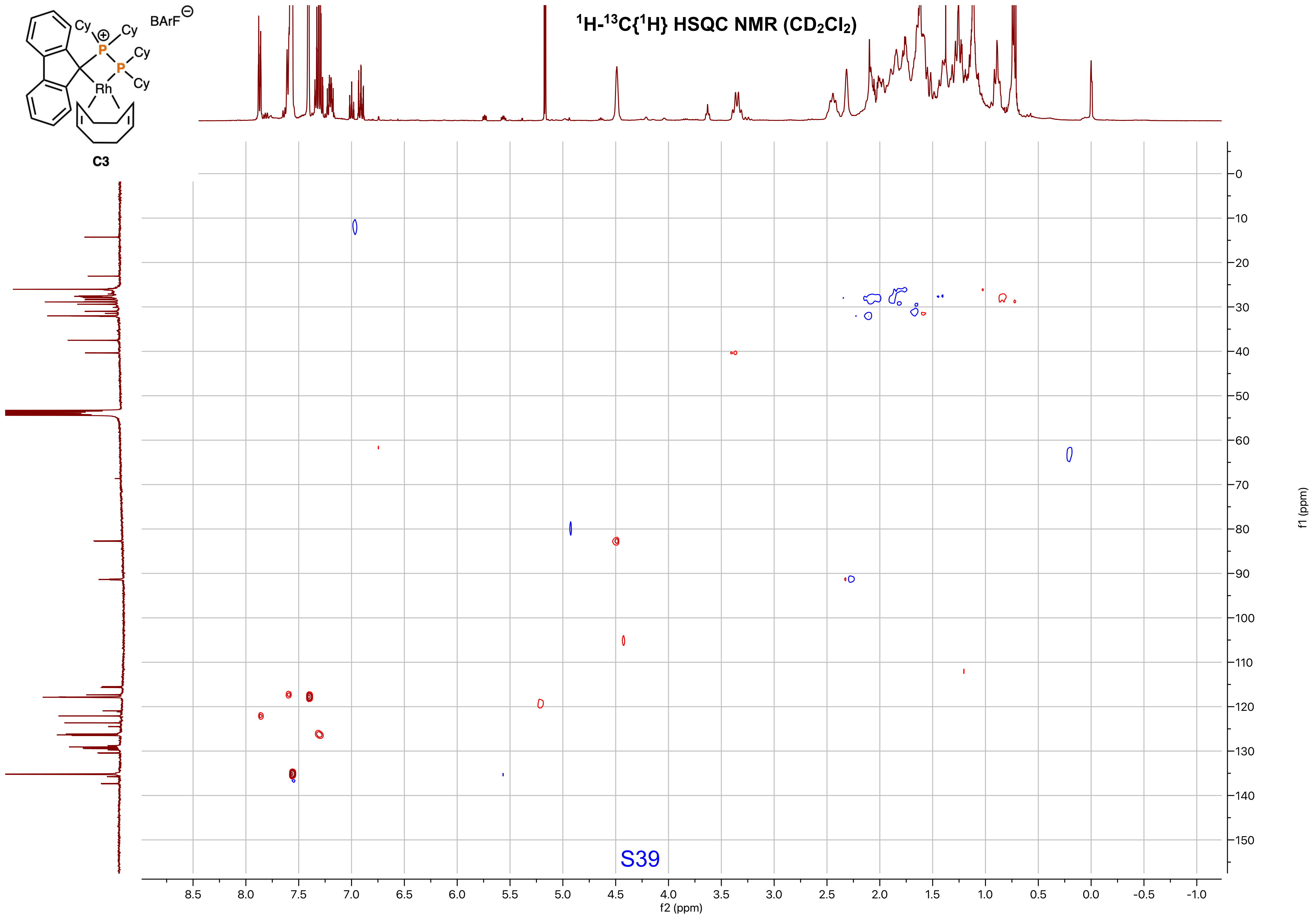

Cy
Cy
Cy
Cy
BArF
P
P
Rh
C3
^{1}H-$^{13}C\{^{1}H\}$ HSQC NMR (CD_2Cl_2)
f1 (ppm)
f2 (ppm)

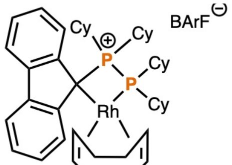

C3

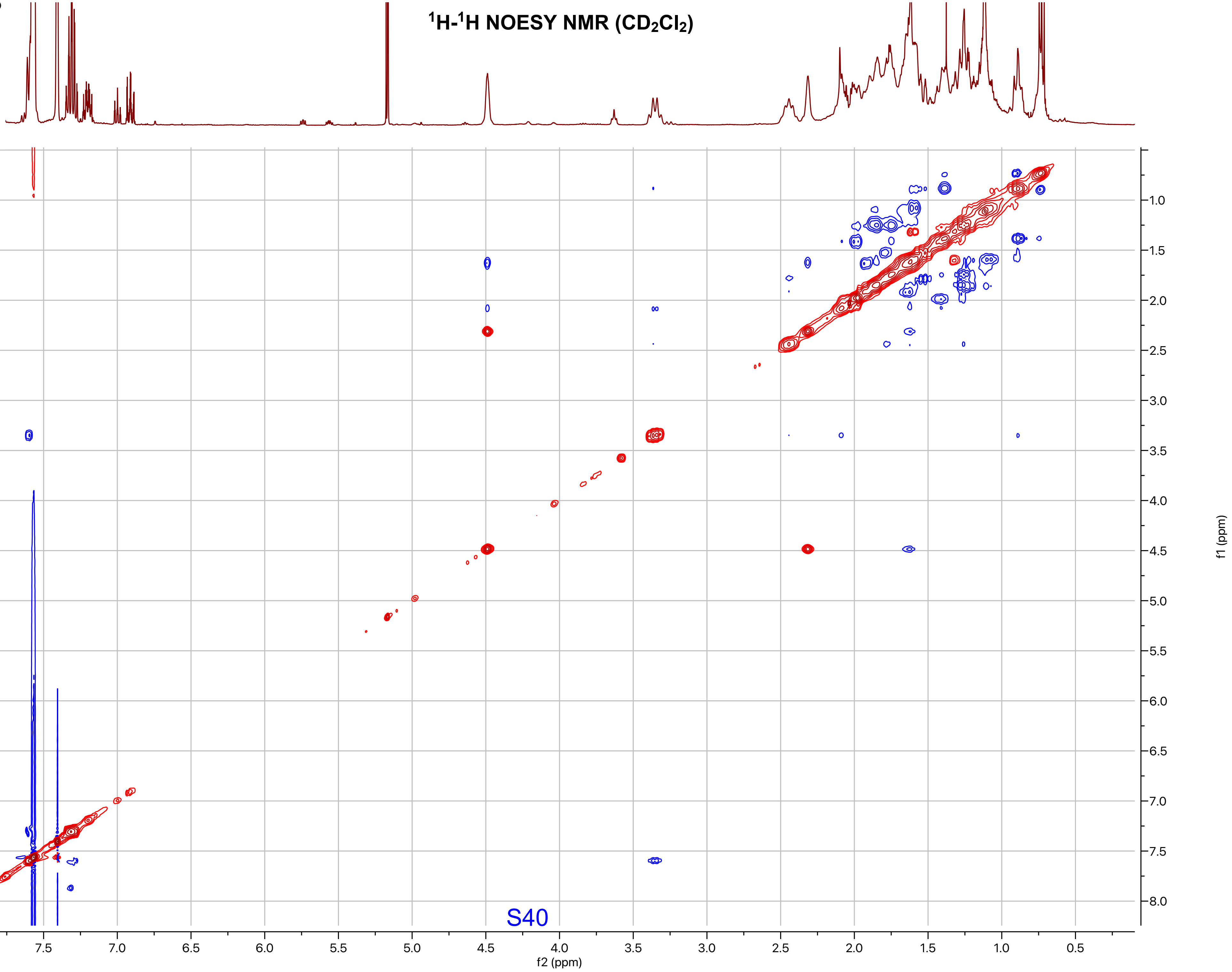

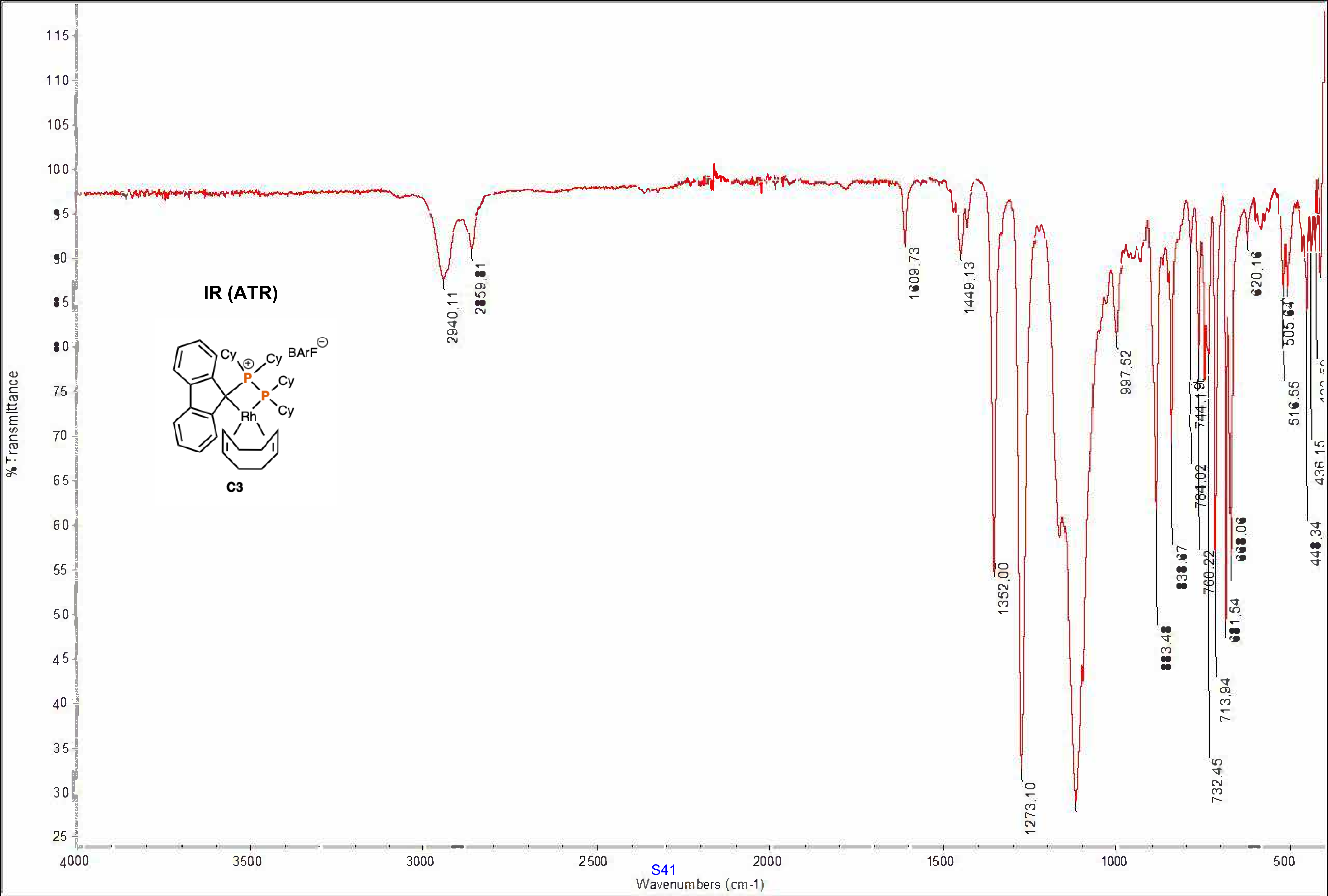

IR (ATR)
Cy
Cy
Cy
Cy
BArF
P
P
Rh
C3
2940.11
1449.13
1352.00
1273.10
997.52
760.22
732.45
713.94
516.55
436.15
%Transmittance
Wavenumbers (cm-1)

HRMS

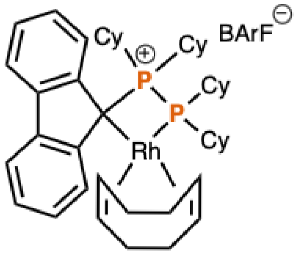
Cy
Cy
BArF
Cy
Cy
P
P
Rh

C3

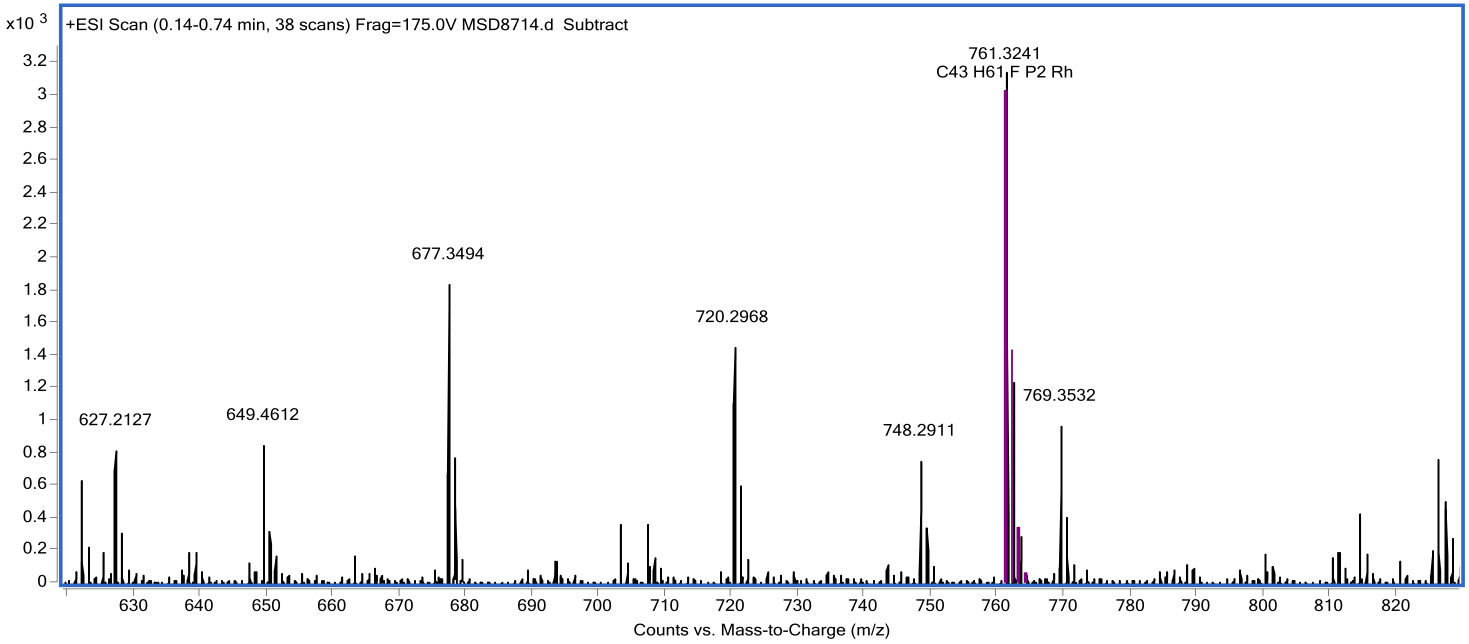
x10 3
+ESI Scan (0.14-0.74 min, 38 scans) Frag=175.0V MSD8714.d Subtract
761.3241
C43 H61 F P2 Rh
677.3494
720.2968
627.2127
649.4612
748.2911
769.3532
Counts vs. Mass-to-Charge (m/z)
